# An integrative insight into the diversity, distribution, and biogeography of the freshwater endemic clade of the *Ponticola syrman group* (Teleostei: Gobiidae) in the Caucasus biodiversity hotspot

**DOI:** 10.1002/ece3.9300

**Published:** 2022-09-15

**Authors:** Fatah Zarei, Hamid Reza Esmaeili, Reza Sadeghi, Ulrich K. Schliewen, Marcelo Kovačić, Keyvan Abbasi, Ali Gholamhosseini

**Affiliations:** ^1^ Ichthyology and Molecular Systematics Research Laboratory, Department of Biology, College of Sciences Shiraz University Shiraz Iran; ^2^ Department of Biology Islamic Azad University Borujerd Iran; ^3^ Department of Ichthyology SNSB‐Bavarian State Collection of Zoology München Germany; ^4^ Natural History Museum Rijeka Rijeka Croatia; ^5^ Inland Waters Aquaculture Research Center, Iranian Fisheries Sciences Research Institute Agricultural Research, Education and Extension Organization Bandar Anzali Iran

**Keywords:** genetic diversification, morphology, otolith shape, *Ponticola iranicus*, *Ponticola patimari*

## Abstract

Freshwater habitats of the Caucasus biodiversity hotspot represent a center of endemism for the gobiid genus *Ponticola* Iljin, 1927. Hitherto, large‐scale molecular studies, owing to restricted taxon and geographical sampling, have failed to give an elaborate picture of diversity and evolutionary history of these species. Here, to contribute to filling this gap, we assessed taxonomic diversity, phylogeography and evolutionary history for the south Caspian populations of *Ponticola* presently classified as *P. iranicus* and *P. patimari*, using an integrative taxonomic approach comprising an entire geographic range sampling, and analyses of mitochondrial DNA haplotypes, the head lateral line system, otolith shape, and meristic and morphometric variation. All freshwater samples of the *P. syrman group* belong to a monophyletic clade with two main subclades: a small subclade confined to the upper Sefidroud sub‐basin including the type locality of *P. iranicus* and a large subclade with three geographically constrained haplogroups (Hg1, Hg2, and Hg3), comprising the rest of the distribution. Hg1 showed an eastern distribution including the type locality of *P. patimari*, while Hg2 and Hg3 are sister groups with central and western‐central distributions, respectively. The freshwater clade diverged from *P. syrman* during the Tyurkyanian low stand (~150 m b.s.l. lasting ~0.1 Myr), while the divergence of *P. iranicus* and *P. patimari* and radiations within *P. patimari* took place during the Bakunian high stand (up to 50 m a.s.l. lasting ~378–480 kya). Species delimitation analyses indicated two distinct species, corresponding to each main subclade. Although the otolith shape and lateral line analyses did not reflect with phylogeographic pattern, PCA and DFA plots of meristic and morphometric data showed a clear separation of the two major subclades corresponding to *P. iranicus* and *P. patimari*, suggesting the presence of significant morphological variation meriting formal taxonomic recognition. Overall, our findings (i) reveal the presence of two freshwater endemic species in the *P. syrman group*, and pending further investigation, hypothesize the presence of a third cryptic species; (ii) revise and document a narrow distributional range and low diversity for *P. iranicus*, in contrast to a wider distributional range and high diversity for *P. patimari*; (iii) suggest that the climatic oscillations of the Pleistocene were associated with the cladogenesis within the *P. syrman group*; and (iv) allowed for the recognition of conservation units and proposition of management measures.

## Graphical abstract

Freshwater habitats of the Caucasus biodiversity hotspot represent a centre of endemism for the gobiid genus *Ponticola* Iljin, 1927. In this paper, we assessed taxonomic diversity, phylogeography and evolutionary history for the south Caspian populations of Ponticola presently classified as *P. iranicus* and *P. patimari*, using an integrative taxonomic approach. Overall, our findings reveal the presence of two freshwater endemic species in the *P. syrman group*, and pending further investigation, hypothesize the presence of a third cryptic species; revise and document a narrow distributional range and low diversity for *P. iranicus*, in contrast to a wider distributional range and high diversity for *P. patimari*; suggest that the climatic oscillations of the Pleistocene were associated with the cladogenesis within the *P. syrman group*; and allowed for the recognition of conservation units and proposition of management measures.

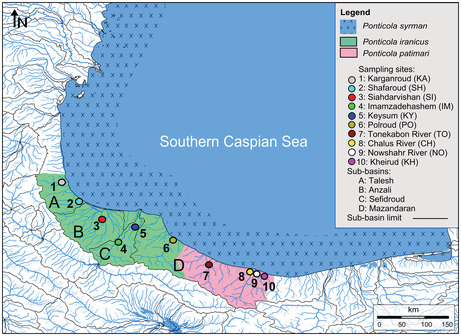

## 1 INTRODUCTION

All species of endemic Ponto‐Caspian gobiids have been described in the genus *Gobius* Linnaeus, 1758. The four subgenera were suggested for relict Sarmatian gobiids of *Gobius* genus by Iljin (1927) (Miller & Vasil'eva, 2003). Berg (1949) separated all these species from *Gobius* into a single genus, *Neogobius* Iljin, 1927, raising the taxon to generic level that covered all subgenera. Later, molecular analyses revealed the paraphyly of *Neogobius sensu* Berg, 1949, and therefore *Ponticola* Iljin, 1927 and *Babka* Iljin, 1927 subgenera (Miller & Vasil'eva, 2003), were also raised to generic rank (Neilson & Stepien, 2009a; Patzner et al., 2011; Stepien et al., 2005; Stepien & Tumeo, 2006).

*Ponticola* Iljin, 1927 represents a monophyletic clade (Neilson & Stepien, 2009a), and endemic gobiid genus to the Black and Caspian Sea basins (Miller, 2003) with 17 valid species (Zarei, Esmaeili, Kovačić, et al., 2022a). In this ecologically diverse group, there are several species which never enter pure freshwaters, but are restricted to the brackish waters of the Black and Caspian seas [i.e., *P. cephalargoides* (Pinchuk, 1976), *P. eurycephalus* (Kessler, 1874), *P. goebeli* (Kessler, 1874), *P. iljini* (Vasil'eva & Vasil'ev, 1996), *P. ratan* (Nordmann, 1840), *P. platyrostris* (Pallas, 1814), and *P. syrman* (Nordmann, 1840)]. *Ponticola kessleri* (Günther, 1861) and *P. gorlap* (Iljin, 1949) are euryhaline and are able to inhabit the sea as well as a wide range of freshwater habitats. In addition, there are several real freshwater endemics including *P. constructor* (Nordmann, 1840), *P. cyrius* (Kessler, 1874), *P. iranicus* Vasil'eva et al., 2015, *P. patimari* Eagderi et al., 2020, *P. rhodioni* (Vasil'eva & Vasil'ev, 1994), *P. rizensis* (Kovačić & Engin, 2008), *P. turani* (Kovačić & Engin, 2008), and *P. hircaniaensis* Zarei, Esmaeili, Kovačić, et al., 2022a, all endemic to small areas in the Caucasus Biodiversity Hotspot (CBH). *Ponticola cyrius* is endemic to the Kura River drainage from headwaters in Turkey down to Azerbaijan, *P. constructor* and *P. rhodioni* are endemic to the south and north of the Bzyb Mountain Range, respectively, *P. rizensis* and *P. turani* are endemic to the İyidere and Aksu streams in Turkey, respectively, and *P. iranicus*, *P. patimari*, and *P. hircaniaensis* are endemic to small areas of the Iranian part of CBH (Eagderi et al., 2020; Kovačić & Engin, 2008; Miller, 2003; Vasil'eva et al., 2015; Zarei, Esmaeili, Kovačić, et al., 2022a).

Molecular clock analysis has suggested that *Ponticola* originated in late Miocene/early Pliocene (ca. 5 Mya; Neilson & Stepien, 2009a), coinciding with the initial separation of the Black and Caspian Sea basins, with subsequent diversifications during the Pliocene and Pleistocene epochs associated with glaciations, fluctuations in water levels, and salinity shifts within the Ponto‐Caspian basin (e.g., Forte & Cowgill, 2013; Krijgsman et al., 2019; Neilson & Stepien, 2009a; Reid & Orlova, 2002), which promoted isolation, adaptation and divergence into localized distinct lineages in many aquatic groups (e.g., Audzijonyte et al., 2006; Brown & Stepien, 2008; Hoyle et al., 2021; Kotlik et al., 2008; Naseka & Bogutskaya, 2009; Parvizi et al., 2018; Sands et al., 2019; Zarei, Esmaeili, Abbasi, et al., 2021a; Zarei, Esmaeili, Schliewen, et al., 2021b). Alternatively, a skeleton and otolith‐based hypothesis in Schwarzhans et al. (2017) suggests that all major endemic Ponto‐Caspian gobiid lineages including *Ponticola* were already present in the Paratethys during the middle Miocene.

The taxonomic composition within *Ponticola* has been variable and frequently uncertain because of mosaic patterns of morphological and karyological features, and presence of distinctive migratory and resident populations. In the south Caspian basin (SCB), for example, Ahnelt and Holčik (1996) collected gobies from four rivers of the Anzali Wetland watershed and identified them as *P. cyrius* and *P. iljini*, both as new for the fish fauna of Iran and for SCB. Neilson and Stepien (2009a) placed *P. iljini* with *P. gorlap* in their revised phylogenetic systematic classification, however, Vasil'eva et al. (2016) proposed the validity of *P. iljini* in a karyological analysis but also restricted its distribution to the west coast of Kazakhstan, northern Caspian Sea. Morphological and karyological examination of *Ponticola* specimens from the Sefidrud and Gisum rivers in Iran by Vasil'eva et al. (2015) identified noticeable differences with other species of *Ponticola* including *P. cyrius*, which led to the description of *P. iranicus*. Phylogenetic and species delimitation analyses of the south Caspian gobiids by Zarei, Esmaeili, Schliewen, et al. (2021b) only supported the presence of two species, *P. gorlap* and *P. iranicus* in the freshwater habitats of the SCB, indicating that the fishes previously identified by Ahnelt and Holčik (1996) as *P. cyrius* and *P. iljini* were in fact conspecific with *P. iranicus* and *P. gorlap*. During the publication of Zarei, Esmaeili, Schliewen, et al. (2021b), however, *P. patimari* was described as a new species from the Kheirud, Chalus, and Tonekabon rivers, all in the middle Mazandaran sub‐basin. It belongs to the *P. syrman group* and is the sister species of *P. iranicus* (Eagderi et al., 2020).

**FIGURE 1:**
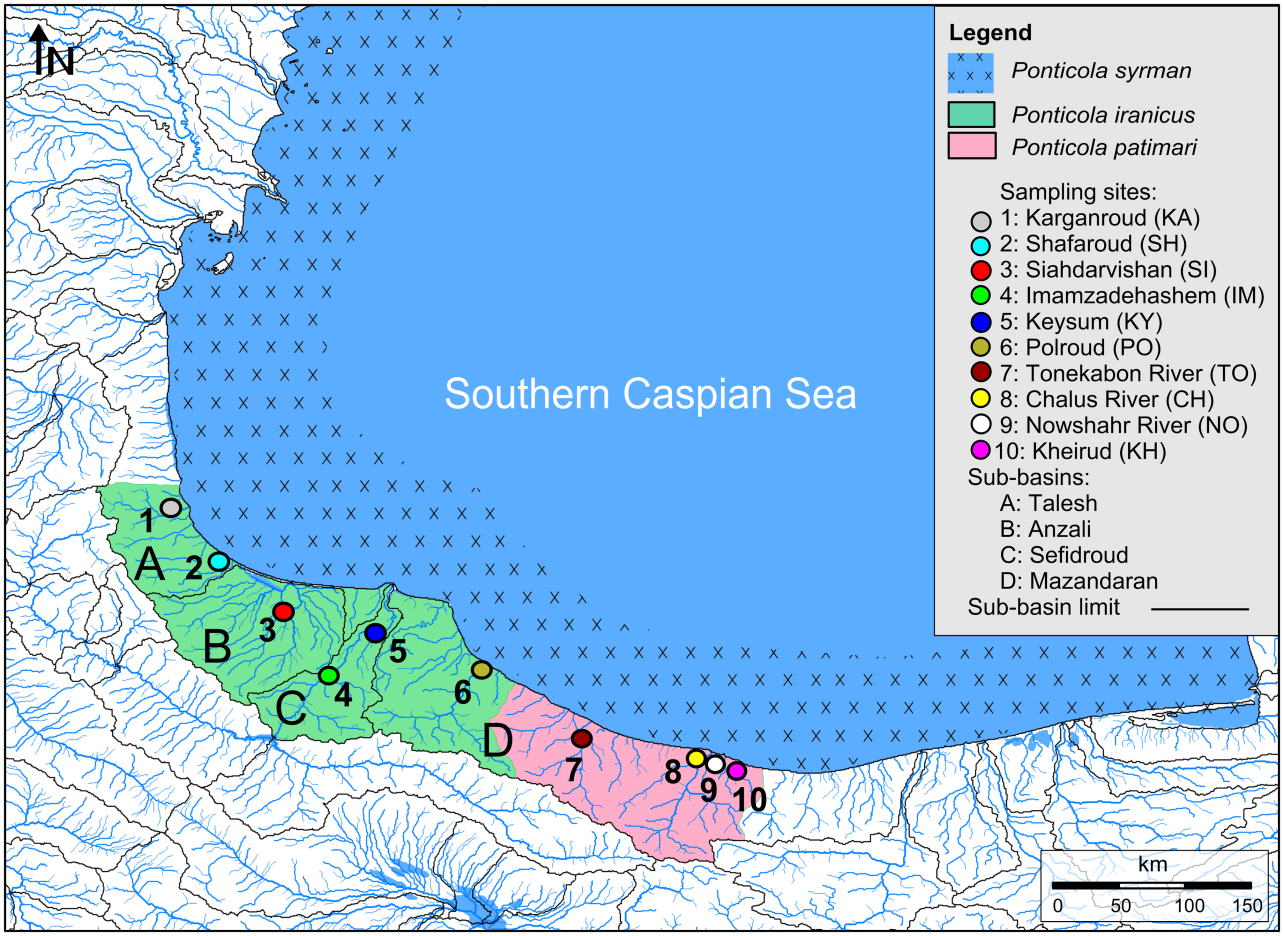
The distribution ranges of *P. iranicus* according to Vasil'eva et al. (2015) and Zarei, Esmaeili, Schliewen, et al. (2021b) (green area), *P. patimari* according to Eagderi et al. (2020) (pink area), and *P. syrman* according to Zarei, Esmaeili, Kovačić, et al. (2022a) and Pinchuk et al. (2003b). The numbered circles refer to the locations of samples used in this study. The map was originally designed using the sub‐basins layer of HydroBASINS 1.0 (Lehner & Grill, 2013) in DIVA‐GIS 7.5 and Surfer 11.

Hitherto, studies have covered major phylogenetic aspects of the Ponto‐Caspian gobiids (e.g., Neilson & Stepien, 2009a; Zarei, Esmaeili, Schliewen, et al., 2021b), however, owing to restricted taxon and geographical sampling, these studies have failed to give an elaborate picture of inter‐ and intraspecific genetic and biogeographic relationships. The diversity, distribution and population structure, historical demography, biology, ecology, and conservation status of the freshwater endemic species of the genus *Ponticola* remain largely undescribed; thus, here we aimed to contribute to filling this gap by studying the freshwater endemic members of the *Ponticola syrman group* across their entire distributional ranges. The *P. syrman group*, as presently understood, comprises a monophyletic clade of three closely related species (Zarei, Esmaeili, Kovačić, et al., 2022a), i.e., *P. syrman*, native to inshore marine and brackish water habitats of the Black Sea, the Sea of Azov, and the Caspian Sea basins (Pinchuk et al., 2003b; Zarei, Esmaeili, Kovačić, et al., 2022a), and *P. iranicus* and *P. patimari*, which are endemic species to the freshwater habitats of the south Caspian Sea basin in northern Iran (Figure 1; Eagderi et al., 2020; Vasil'eva et al., 2015; Zarei, Esmaeili, Schliewen, et al., 2021b; Zarei, Esmaeili, Kovačić, et al., 2022a). We therefore (i) assess taxonomic diversity, and species level status of different south Caspian populations of *Ponticola* presently classified as *P. iranicus* and *P. patimari* within an integrative taxonomic framework comprising an entire geographic range sampling, molecular analysis, cephalic lateral line system, otolith shape, meristic and morphometric analyses, (ii) infer phylogeographic patterns and their evolutionary history and historical demography, and (iii) we discuss the implications of our results regarding conservation.

## 2 METHODS

### 2.1 Study area, taxon sampling, and preservation

Current literature suggests that *Ponticola iranicus sensu* (Vasil'eva et al., 2015) is distributed in the Talesh, Anzali, Sefidroud and west Mazandaran sub‐basins (Karganroud to Polroud), while *P. patimari sensu* (Eagderi et al., 2020) is distributed in the middle Mazandaran sub‐basin (see Figure 1; Eagderi et al., 2020; Vasil'eva et al., 2015; Zarei, Esmaeili, Schliewen, et al., 2021b). Accordingly, new samples were collected from seven localities of four hydrographic sub‐basins in the SCB: Karganroud (KA) and Shafaroud (SH) from the Talesh sub‐basin, Siahdarvishan (SI) from the Anzali sub‐basin, Imamzadehashem (IM) and Keysum (KY) from the Sefidroud sub‐basin, and Polroud (PO) and the Chalus River (CH) from the Mazandaran sub‐basin (see Figure 1, Table 1). Three additional *P. patimari* samples were included from Eagderi et al. (2020) and Zarei, Esmaeili, Schliewen, et al. (2021b), i.e., the Kheirud (KH), Tonekabon (TO), and Nowshahr (NO) rivers from the Mazandaran sub‐basin (Figure 1, Table 1). Specimens were collected using an electro‐fishing device. Quinaldine sulfate (2‐methylquinoline sulfate) was used as anesthetic. The right pectoral fin of each specimen was fixed in 96% ethanol for molecular analysis, while the voucher specimens were fixed in 70% ethanol or 10% formaldehyde for otolith and morphological analyses. These specimens are deposited in the Zoological Museum of Shiraz University, Collection of Biology Department, ZM‐CBSU (Table 1).

**TABLE 1:** Studied samples of *P. iranicus* and *P. patimari* (see the initial and revised classifications) from the SCB in the molecular (COI), otolith outline, meristic, morphometric and head lateral line analyses

Sub‐basin	Sample	Code	Coordinates	Species		COI analysis			Otolith outline analysis			Morphological analysis		
				Initial classification	Revised classification/lineage	*N*	Molecular ID	GenBank No.	*N*	ZM‐CBSU	SL range	*N*	ZM‐CBSU	SL range
Talesh	Karganroud	KA	37.80, 48.89	*P. iranicus*	*P. patimari*/Hg3	2	S004–S005	ON853683–ON853684	–	–	–	–	–	–
	Shafaroud	SH	37.57, 49.13	*P. iranicus*	*P. patimari*/Hg3	15	GO1997^a^–GO1999^a^, E001–E003, E006, E009, E016–E017, E019, S001–S002, S006–S007	MW393611^a^–MW393613^a^, ON853728–ON853739	46	24_01–16, 24_19–21, 24_30–31, 24_34–43, 24_46–47, 24_52–55, 24_60–68	56.45–73.97	23	S090‐1–S090‐23	39.3–96.5
Anzali	Siahdarvishan	SI	37.35, 49.42	*P. iranicus*	*P. patimari*/Hg3	18	P2819^a^–P2820^a^, E026–E027, E030–E031, E034–E035, E037–E038, E041–E046, E048, E050	MW393605^a^–MW393606^a^, ON853698–ON853713	48	25_01–48	55.91–74.56	23	S080‐3–S080‐25	39.6–66.1
Sefidroud	Imamzadehashem (Sefidroud upstream)	IM	37.02, 49.64	*P. iranicus*	*P. iranicus*	14	E097–E103, E105, E107–E108, M2170, ICH‐059656^b^–ICH‐059657^b^, ICH‐059662^b^	ON853714–ON853727	19	21_01–04, 21_06–20	58.00–68.74	23	S087‐1–S087‐23	40.0–82.0
	Keysum (Sefidroud downstream)	KY	37.23, 49.85	*P. iranicus*	*P. patimari*/Hg2–3	10	E109–E118	ON853757–ON853766	11	22_01–11	58.56–67.08	–	–	–
Mazandaran	Polroud	PO	37.09, 50.37	*P. iranicus*	*P. patimari*/Hg2–3	18	P2780^a^, E051, E054, E056–E058, E060–E071	MW393609^a^, ON853740–ON853756	29	23_01–29	55.85–72.18	13	S091‐1–S091‐13	53.4–75.7
	Tonekabon	TO	36.73, 50.84	*P. patimari*	*P. patimari*/Hg1	1	1089‐47v^a^	MW256420^a^	–	–	–	–	–	–
	Chalus River	CH	36.65, 51.41	*P. patimari*	*P. patimari*/Hg1	14	E072–E074, E076–E080, E090–E091, E093–E095, 1086‐45t^a^	MW256419^a^, ON853685–ON853697	60	20_01–60	57.68–69.24	21	S089‐1–S089‐21	37.7–98.1
	Nowshahr River	NO	36.64, 51.48	*P. patimari*	*P. patimari*/Hg1	1	P2779^a^	MW393610^a^	–	–	–	–	–	–
	Kheirud River	KH	36.61, 51.56	*P. patimari*	*P. patimari*/Hg1	2	1087‐46u1^a^, 1087‐46u2^a^	MW256416^a^–MW256417^a^	–	–	–	4	S088‐1–S088‐4	45.6–57.4
						95			213			107		

*Note*: Specimens used in the classical otolith morphometric and shape analysis are mentioned in the text.

Abbreviation: *N*, number of fish.

^a^ Data mined from Zarei, Esmaeili, Schliewen, et al. (2021b) or Eagderi et al. (2020).

^b^ Data from ZFMK; SL, standard length in mm.

### 2.2 DNA extraction and PCR

Genomic DNA was extracted from fin samples preserved in 96% ethanol using a salt method protocol following Bruford et al. (1992). PCR amplification of the standard vertebrate DNA barcode region, the mitochondrial cytochrome c oxidase I (COI) was performed using the primer pairs, FishF1 and FishR1 (Ward et al., 2005), or FISH‐BCL and FISH‐BCH (Baldwin et al., 2009). The 25 μl PCR reaction mixes included 12.5 μl of a ready 2X Taq PCR Master Mix (Parstous^TM^), 0.5 μl of each primer (10 pmol/μl), 6 μl of the target DNA, and 5.5 μl DNase‐free distilled water. The PCR conditions were initial denaturation for 3 min at 94°C followed by 35 cycles of 94°C for 45 s, 52–56°C for 45 s, and 72°C for 45 s, and final extension for 5 min at 72°C. PCR products were purified using ExoSAP‐IT^TM^ and sequenced by the Niagene Lab. (Tehran, Iran) using Applied Biosystems^TM^ BigDye^TM^ Terminator v3.1 Cycle Sequencing Kit on an Applied Biosystems^TM^ ABI PRISM 3730xl.

In addition to the newly sequenced material, our molecular investigation also analyzed the following COI datasets (Tables 1 and S1): (i) *P. iranicus* COI barcodes mined from Zarei, Esmaeili, Schliewen, et al. (2021b), collected from Shafaroud (SH), Siahdarvishan (SI), Polroud (PO) and the Nowshahr River (NO); (ii) COI barcodes of the FREDIE project (Freshwater Diversity Distribution for Europe) deposited by Zoologisches Forschungsmuseum Alexander Koenig (ZFMK), collected from Sefidroud at Imamzadehashem (IM) and classified as *P. iranicus*; (iii) GenBank deposited COI barcodes of *P. patimari* mined from the species original description by Eagderi et al. (2020), collected from Kheirud (KH), Tonekabon (TO), and the Chalus (CH) rivers; and (iv) archived COI barcodes of different *Ponticola* species mined from GenBank [derived from Neilson & Stepien, 2009a, and Zarei, Esmaeili, Schliewen, et al., 2021b] and ZFMK (Table S1). In general, COI does not give enough resolution for deep phylogenetic analyses, but for the taxonomic scope presented here it provides sufficient phylogenetic resolution for a first step for integrative taxonomy.

### 2.3 Molecular data analysis

COI sequences (659 bp) were edited with BioEdit 7.0.4 (Hall, 1999), and aligned using ClustalW algorithm in Mega 7.0 (Kumar et al., 2016). All newly obtained sequences are deposited in GenBank (Table 1). Variation was estimated as number of polymorphic sites (s), number of haplotypes (H), haplotype diversity (Hd), nucleotide diversity (π), and average number of pairwise nucleotide differences (k) using Arlequin 3.5.2.2 (Excoffier & Lischer, 2010). Historical demographic patterns were investigated using (a) neutrality tests including Tajima's D (Tajima, 1989) and Fu's Fs (Fu, 1997) in Arlequin based on 10,000 permutations, and R2 (Ramos‐Onsins & Rozas, 2002) in DnaSP 6 (Librado & Rozas, 2009), and (b) mismatch distribution (MMD; Rogers & Harpending, 1992) in Arlequin. To discover deviation from the expectations of the neutral theory (Kimura, 1968), the applied neutrality tests differ in their approach: Tajima's D and R2 use the mutation frequency information, whereas Fu's Fs uses the haplotype distribution data.

Phylogeographic depth and genealogical relationships between haplotypes were depicted using the median‐joining (MJ) algorithm in PopART 1.7 (Leigh & Bryant, 2015). Arlequin was used to estimate the pairwise Fst values (Wright, 1965) among samples. Analysis of molecular variance (AMOVA; Excoffier et al., 1992) was used to estimate the partitioning of genetic variance among groups, among samples within groups, and within samples. Geographic Distance Matrix Generator 1.2.3 (Ersts, 2012) was used to estimate a geographic distance matrix. Mantel test (Mantel, 1967) was used to discover possible effect of isolation‐by‐distance (IBD; Slatkin, 1993) with Vegan 2.0 (Dixon, 2003) in R 4.0.5 (Ihaka & Gentleman, 1996). The saturation test of Xia et al. (2003) in DAMBE 7 (Xia, 2018) was used to test the nucleotide substitution saturation in the sequence under study. The best‐fit nucleotide substitution model for the dataset was estimated based on the Bayesian information criterion (BIC) in jModelTest 2.1.3 (Darriba et al., 2012). A Maximum Likelihood (ML) phylogeny using 5000 bootstrap replicates (fast bootstrap) was generated in RAxML 7.2.5 (Stamatakis, 2006).

To delineate putative species, (i) a statistical parsimony (SP) network based on a 95% connection probability threshold was estimated in TCS 1.21 (Clement et al., 2000) and (ii) an Assemble Species by Automatic Partitioning analysis (ASAP; Puillandre et al., 2021) was performed through its web interface (https://bioinfo.mnhn.fr/abi/public/asap/asapweb.html) using Kimura (K80) ts/tv (=0.2). Furthermore, cutoff value of 2% K2P sequence divergence for COI (Ward, 2009) was used as indicator of distinct species.

BEAST 1.7.4 (Drummond et al., 2012; Drummond & Rambaut, 2007) was used for divergence time estimations using limited haplotypes from different lineages to avoid polytomies. Bayesian inference of phylogeny (BI) was performed in BEAST, run using an uncorrelated lognormal relaxed clock model and a birth‐death speciation prior. The phylogeny was calibrated with a legacy date of 4.07 Mya at the base of *Ponticola* (mined from Neilson & Stepien, 2009a). A secondary calibration of 0.9 Mya was assigned to the node subtending *P. syrman* + *P. iranicus* + *P. patimari* (based on Zarei, Esmaeili, Schliewen, et al., 2021b) and the analysis was run in four independent runs of 100,000,000 generations, with trees sampled every 1000 generations; the first 10% were discarded as burn‐in. Finally, convergence and effective sampling sizes (ESS) were checked in Tracer 1.6 (Rambaut et al., 2018), and a maximum clade credibility consensus tree was built in Tree Annotator 1.8.2 (Drummond et al., 2012).

### 2.4 Otolith SEM imaging and morphometric analysis

**FIGURE 2:**
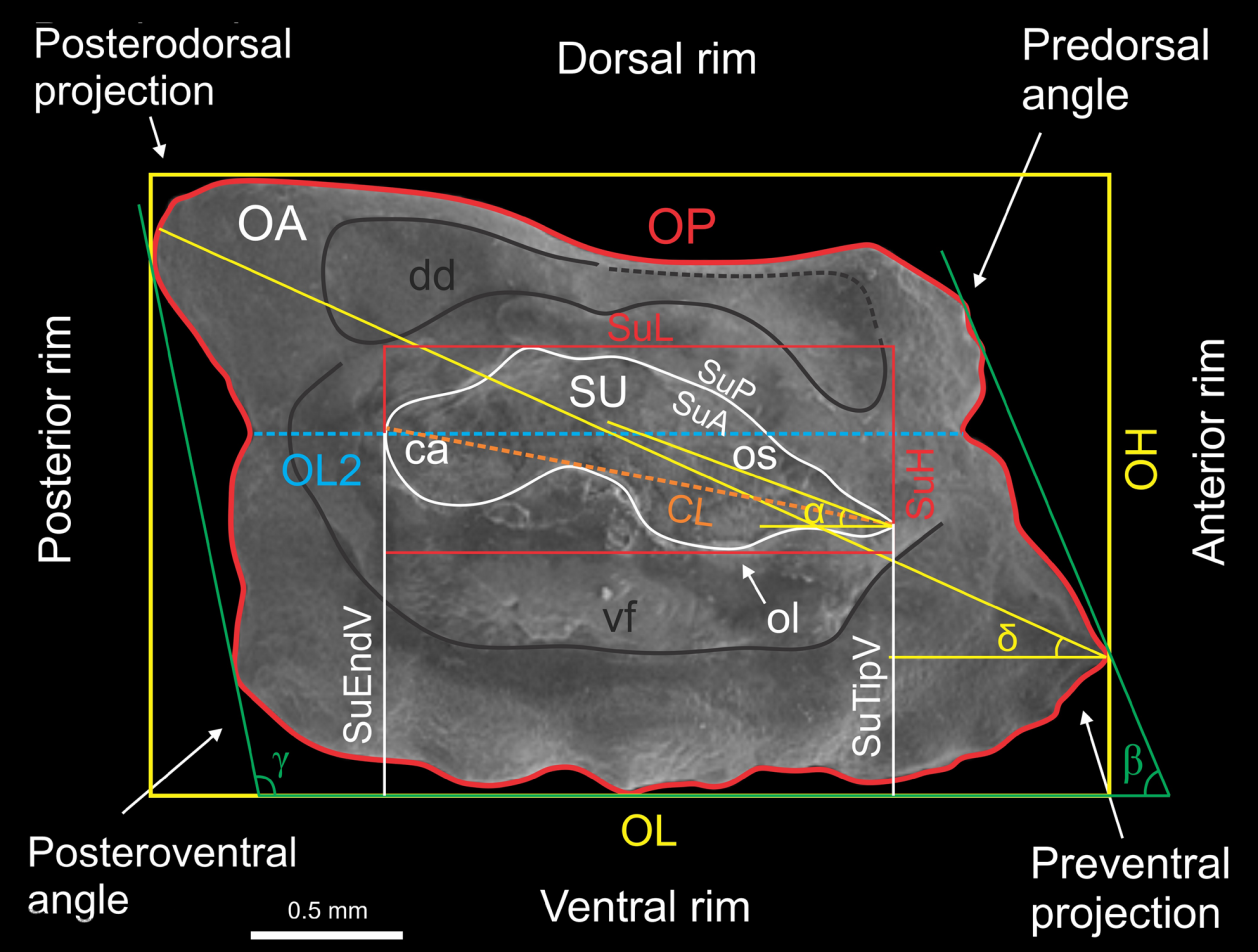
SEM photo of the left sagittal otolith inner face of *P. iranicus sensu* Vasil'eva et al. (2015) [ZM‐CBSU 44, 72.8 mm SL, Sefidroud at Imamzadehashem (IM)], showing the terminology of characters and otolith outline (red line). Terminology: OL, maximal otolith length; OL2, minimal otolith length measured at maximum ingression of concavity of posterior rim; OH, maximal otolith height; OA, otolith area; OP, otolith perimeter; SU, sulcus; ca, cauda; os, ostium; CL, colliculum length measured along its axis; SuL, sulcus length; SuH, sulcus height; SuP, sulcus perimeter; SuA, sulcus area; SuTipV, distance from sulcus tip to the ventral margin; SuEndV, distance from sulcus end to the ventral margin; dd, dorsal depression; vf, ventral furrow; si, subcaudal igum; α, inclination angle of ostium measured from tip of ostium through midpoint of sulcus height at collum; β, inclination angle of anterior rim; γ, inclination angle of posterior rim; δ, inclination of line connecting preventral angle with tip of poserotdorsal projection.

The head region of *P. iranicus* specimens from Sefidroud at IM (ZM‐CBSU 37–40, 141 & 144, 6 spec., 63.8–76.7 mm SL) and *P. patimari* specimens from NO (ZM‐CBSU P1–P4 & P6, 5 spec., 51.69–65.64 mm SL) were dissected under a Zeiss^TM^ Stemi SV6 stereomicroscope. The left sagittae (= left saccular otolith) was extracted using fine tweezers, cleaned by incubation in 1% KOH solution (3 min), washed in distilled water and then dried at room temperature. Otoliths were coated with gold, and scanning electron microscope (SEM) images were taken using a TESCAN^TM^ VEGA3 housed at the Shiraz University's Central Lab. Morphological terminology for the sagittae follows Gierl et al. (2018) and Schwarzhans et al. (2020), and is shown in Figure 2. Otolith measurements were taken in ImageJ 1.52a (Figure 2): OL, maximal otolith length; OL2, minimal otolith length measured at maximum ingression of concavity of posterior rim; OH, maximal otolith height; CL, colliculum length measured along its axis; SuL, sulcus length; SuH, sulcus height; OP, otolith perimeter (in mm); OA, otolith area (in mm^2^); SuP, sulcus perimeter (in mm); SuA, sulcus area (in mm^2^); SuTipV, distance from sulcus tip to the ventral margin and; SuEndV, distance from sulcus end to the ventral margin. All distances were measured to the nearest 0.001 mm. The measurements were used to calculate 24 otolith variables [following Tuset et al., 2003; Reichenbacher et al., 2007; Gierl et al., 2018, and Schwarzhans et al., 2020], based on the ratios between the individual otolith measurements: OL/OH (= aspect ratio, ASr), OP/OL, OP/OH, SuA/OA, SuP/OP, SuP/SuTipV, SuP/SuEndV, SuL/OL, SuL/OH, SuL/SuH, SuL/SuTipV, SuL/SuEndV, SuL/OP, SuL/SuP, SuH/OL, SuH/OH, SuH/SuTipV, SuH/SuEndV, SuH/OP, SuH/SuP, SuTipV/OP, SuTipV/SuEndV, SuEndV/OP, and OL2/CL. The otolith measurements and calculated variables are presented for all specimens in Table S2.

Following Schwarzhans et al. (2020), four inclination angles were measured for each otolith (Figure 2): α, inclination angle of ostium measured from ostium tip through midpoint of sulcus height at collum; β, inclination angle of anterior rim; γ, inclination angle of posterior rim; δ, inclination of line connecting preventral angle with tip of posterodorsal projection. All measurements are presented in Table S2. In addition to aspect ratio [ASr; larger ASr values represent more elongation (Ponton, 2006)], another three otolith shape indices were calculated following Ponton (2006) (Table S2): (i) roundness (ROx = 4OA/πOL2), describes the ratio between the actual area and the area of a circle of the same length; it is larger when the otolith shape is more circular; (ii) rectangularity [REx = OA/(OL × OH)], describes the variations of length and width with respect to the area with 1 being a perfect square and <1 being a nonsquare; and (iii) ellipticity [ELx = (OL − OH)/(OL + OH)], showing the proportional change in the short and long axes from 0.0 (a perfect round shape) to close to 1.0 (a spindle shape).

Otolith variables, inclination angles and shape indices were analyzed using IBM SPSS Statistics 26.0 (IBM Corp., 2019). Normal distribution of these variables for each species was examined with the Shapiro–Wilk test (*p* > .05), which indicated that all variables were normally distributed. Univariate analysis of variance (ANOVA) with Tukey's HSD and Dunnett T3 post‐hoc test [depending on homogeneity of variances (Levene's test, *p* > .05)] was used to test the significance of variables, inclination angles, and shape indices differences among the otoliths of *P. iranicus* and *P. patimari*.

### 2.5 Otolith outline analysis

The left sagittal otolith of 213 specimens from seven south Caspian localities (Table 1) were extracted using fine tweezers, cleaned and washed in distilled water, dried at room temperature, then placed on a dark plate and digital images at 4× magnification were taken using a 14MP Industrial Microscope Camera 180× equipped with an S‐EYE 1.2.4.128 image processing system. To measure otolith shape variation, we used statistical functions in R 4.0.5 (Ihaka & Gentleman, 1996) using the packages shapeR 0.1‐5 (Libungan & Pálsson, 2015), vegan 2.5‐7 (Oksanen et al., 2020), ipred 0.9‐12 (Peters et al., 2021), and MASS 7.3‐54 (Ripley et al., 2021). The otolith images were read into R. ShapeR analyzes otolith shape by extracting otolith outlines from these images. A matrix of coordinates (*x*, *y*) from all otolith outlines was calculated. Evenly spaced radii with length as a univariate shape descriptor were drawn from the otolith centroid to its outline. Using the wavelet transformation on radii, the wavelet coefficients were extracted with wavethresh 4.6.8 (Nason, 2016). In order to remove the influence of allometric growth, wavelet coefficients that showed significant (*p* < .05) interaction between samples and SL were excluded (Libungan et al., 2015), and the remaining coefficients were imported into the R statistical packages. The mean otolith shapes for samples were plotted based on wavelet coefficients. To determine areas of otolith shape variation, mean shape coefficients and their standard deviations were plotted against the angle of the otolith outline using wavelet transform with gplots 3.1.1 (Warnes et al., 2020). Because the proportion of variation among groups (intraclass correlation) was more informative to measure between‐population differences, intraclass correlation was estimated along the otolith outline.

The radii length was employed to test for the significance of differences between samples using an ANOVA‐like permutation test (1000 permutations) in vegan. To investigate the statistical significance of otolith shape difference between males and females, sex‐related stability of otolith shape was analyzed in shapeR. The standardized wavelet coefficients were transformed into principal coordinates and subjected to canonical analysis (CAP: Canonical Analysis of Principal coordinates) to assess the variation in otolith shape among the samples. The result was compared among samples using the function capscale in vegan. The wavelet coefficients were further employed for cluster visualization of the CAP results in two discriminating axes (CAP1, CAP2). The CAP outcome was used as input for a dendrogram based on the Squared Euclidean Dissimilarity Distance in PAST 3.03 (Hammer et al., 2001). Using a leave‐one‐out cross‐validation scheme, the classification success into groups was investigated. Accordingly, using the functions *errorest* and *lda* in ipred and MASS, Linear Discriminant Analysis (LDA) on the standardized wavelet coefficients was conducted to show classification of individuals to original samples with cross‐validation estimation. A matrix of average Euclidean distances among samples based on otolith shape (CAP1 and CAP2) was estimated. Applying the Mantel test (Pearson test, 1000 permutations) as implemented in vegan with R, we tested the hypothesis of regional otolith shape differentiation under IBD.

### 2.6 Meristic and morphometric analysis

Morphometric methods represent a composite of Schliewen and Kovačić (2008) and Miller (2003). Eight meristic and 41 morphometric variables were recorded for 107 specimens from six south Caspian localities (Table 1): D1, first dorsal fin elements; D2, second dorsal fin elements; A, anal fin elements; P, pectoral fin rays; C, caudal fin rays; PD, predorsal scales; TR, scales in transverse series; LL, scales in lateral series; in D2 and A counts the last bifid ray is counted as one. SL, standard length (measured from the median anterior point of the upper lip to the base of the caudal fin); Cl, caudal fin length; D1I, first dorsal fin 1st spine length; D1II, first dorsal fin 2nd spine length; D1III, first dorsal fin 3rd spine length; D2I, second dorsal fin 1st spine length; D2h, longest second dorsal fin ray; D1b, first dorsal fin base; D2b, second dorsal fin base; Ab, anal fin base; Aw, body width at the anal fin origin; Ad, body depth at the anal fin origin; CP, caudal peduncle length; CPd, caudal peduncle depth; lapc, width of caudal peduncle at anal fin; Pl, pectoral fin length; SN/A, preanal distance; SN/AN, snout to anus; SN/D1, snout to D1 origin; SN/D2, snout to D2 origin; SN/V, snout to pelvic fin origin; V/A, pelvic fin origin to anal fin origin; V/AN, pelvic fin origin to anus; Vl, pelvic fin length; A1I, 1st anal spine length; Vd, body depth at the pelvic fin origin; HL, head length; Hdn, head depth at nape; Hde, head depth at eye; Hw, head width; E, eye diameter; I, interorbital width; CHd, cheek depth; SN, snout length; LPd, lateral preorbital depth; ULl, upper lip length; Lam, mouth width; AULw, anterior upper lip width; ULw, maximum upper lip width; and PO, postorbital length.

Body shape variation in gobies is allometric, i.e., it is strongly correlated with size. Thus, the size effect was eliminated by the method outlined by Elliott et al. (1995): Mc = M × (Ls/SL)^b^ where Mc is the size adjusted measurement, M is the observed measurement, Ls is the overall mean of the standard length for all fish from all samples in each sampling site, SL is the standard length of the fish, and b is the slope of the regression of log M on log SL, using all specimens. Since SL was used to standardize other parameters, it was removed and not transformed. All data and statistical analyses were carried out using IBM SPSS Statistics 26.0.

One‐way analysis of variance (ANOVA), was conducted to distinguish the significant differences among the observed lineages (see molecular results) based on the size adjusted morphometric and meristic data. Likewise, if significant differences were observed, Tukey's HSD comparison tests were done for pairwise comparisons between lineages. Multivariate analysis of variance (MANOVA) was used to test significant overall difference between lineages.

Principal component analysis (PCA) was performed on all size‐adjusted morphological data using the covariance matrix to identify the variables that contribute the most to the differences and to compare overall morphological patterns among the lineages. Prior to PCA, Bartlett's test of sphericity and the Kaiser–Meyer–Olkin (KMO) measure of sampling adequacy were used to validate the use of the PCA to reduce the size‐adjusted morphometric and meristic data to a few more easily interpretable components. KMO values between 0.8 and 1 indicate the sampling is adequate. Bartlett's test of sphericity (χ^2^ = 4504.68, *p* < .001) was significant and therefore revealed that non‐zero correlations existed at a probability level <.05, and the value of KMO coefficient of sampling adequacy for overall matrix was 0.82. The results of KMO and Bartlett's suggest that the sampled data are appropriate to proceed with a PCA procedure.

To study the distribution of the measured specimens in a multivariate morphometric space, discriminant function analysis (DFA) was performed on the size‐adjusted morphometric and meristic data to extract the most important characters for differentiating lineages, using the *F*‐value criterion. DF scores 1 and 2 were selected and plotted in the discriminant space. Also, group centroids were produced by DFA to visualize associations between lineages. Classification success was taken from DFA to correct assignment of individual fish to original lineages. The size‐adjusted morphometric and meristic data were used as input for a dendrogram based on the Squared Euclidean distance as a measure of dissimilarity.

### 2.7 Head canals and sensory papillae pattern

The sensory canals, pores, and sensory papillae patterns of the head lateral line system provide important diagnostic characters to distinguish goby species (Kovačić, 2020). The head lateral line systems of the samples were checked and mapped using a Zeiss^TM^ Stemi SV6 stereomicroscope. When necessary, the specimens were stained in 2% KMnO_4_ solution for 5 s, which allowed a better examination of sensory papillae rows. Drawing of the head lateral line system was prepared with CorelDRAW® Graphics Suite 2020 (https://www.coreldraw.com/). The terminology of papillae rows, and of canal pores follows Miller (1986) based on Sanzo (1911) and Miller (2003). Canals: AOC, POC, anterior and posterior oculoscapular canals, respectively; PC, preopercular canal. Pores are marked with Greek lettering (Miller, 1986; Sanzo, 1911).

## 3 RESULTS

### 3.1 Genealogy, species delimitation, phylogeography, and divergence time estimation

**FIGURE 3:**
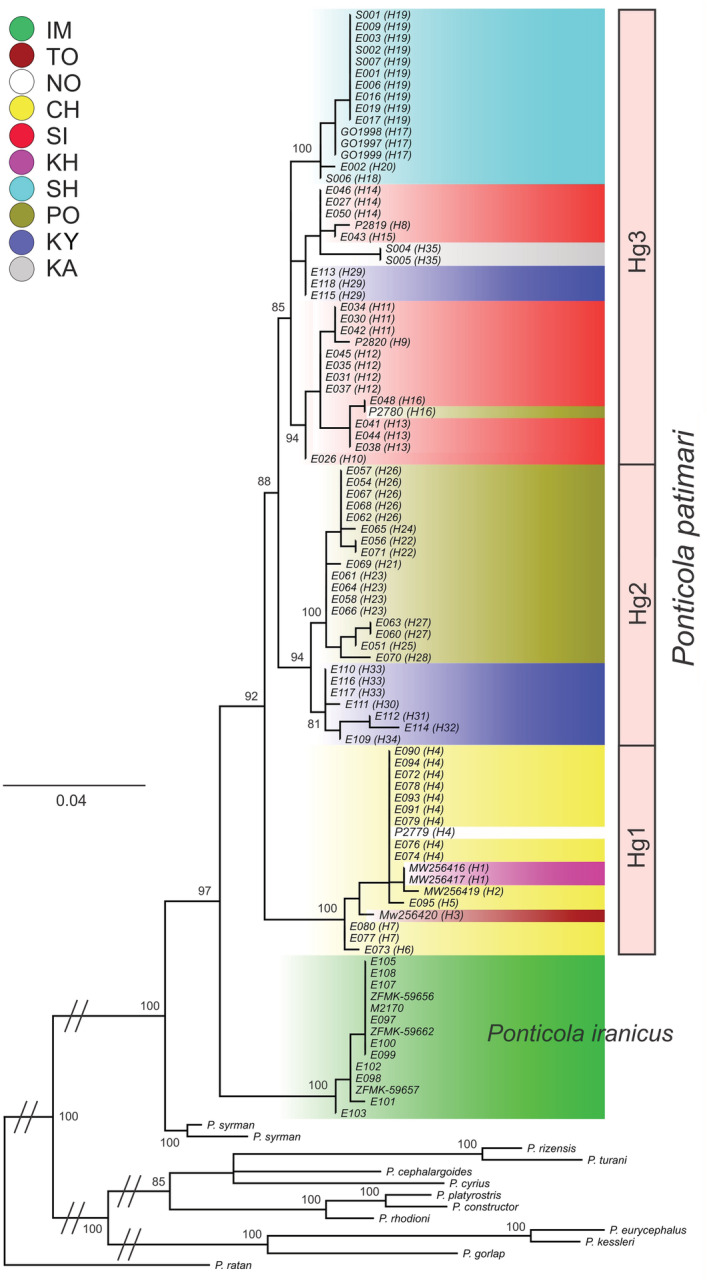
ML phylogeny of *Ponticola* based on mitochondrial COI (108 individuals), with focus on the *P. syrman group*. New sequences produced in the context of this study are shown within the colored clades. The new limits set for *P. iranicus* and *P. patimari* on this tree are based on this phylogeny and the results derived from the species delimitation analyses (i.e., SP, ASAP, and 2% K2P new species threshold; the initial assignment of individuals and samples are presented in the text and Table 1). The information in the parentheses indicates the haplotype. The numbers at the nodes represent ML bootstrap values (BP). Double bars on some branches indicate that those branches have been reduced in length and are not proportional to the scale

**FIGURE 4:**
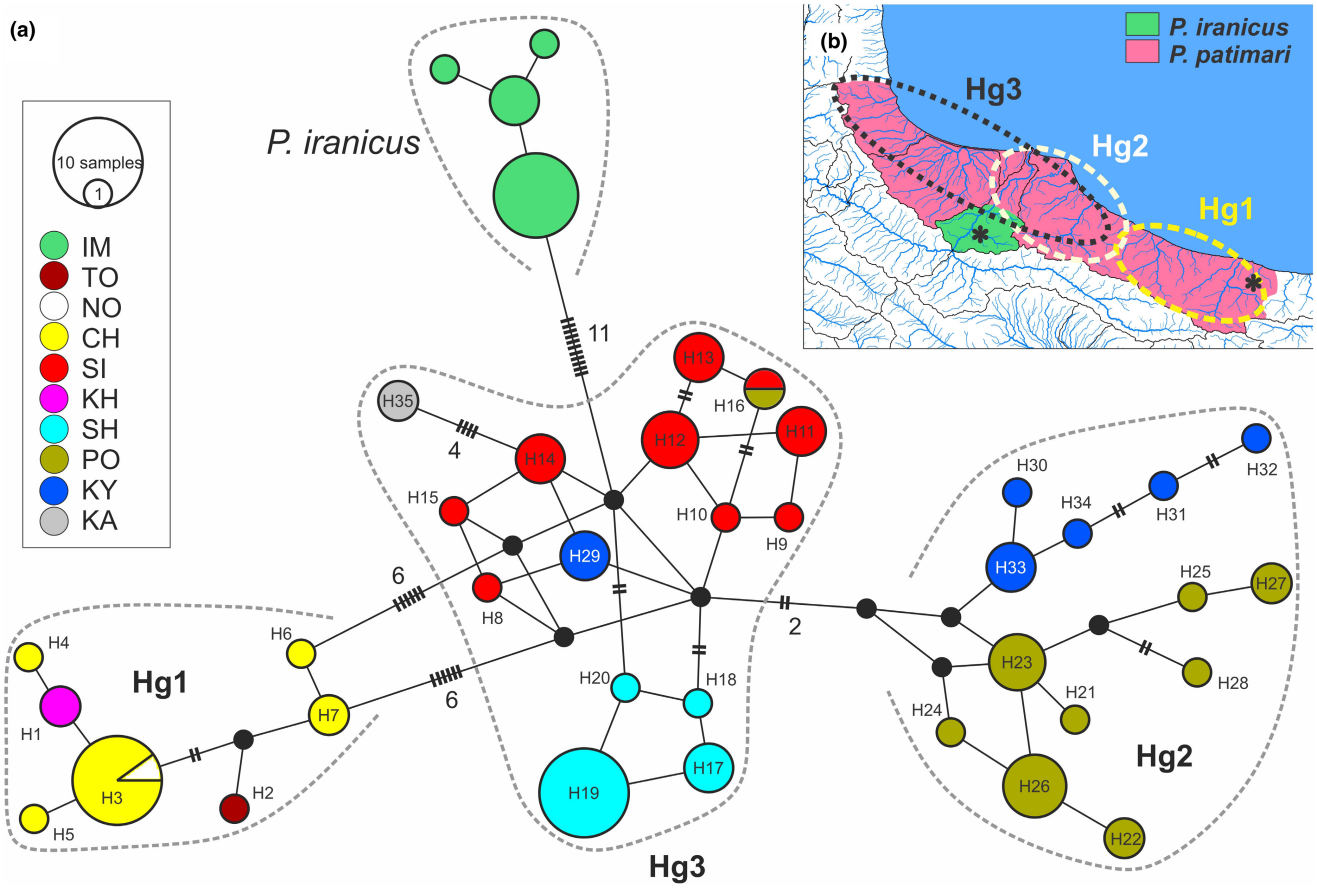
(a) MJ haplotype network for the mitochondrial haplotypes observed in *P. iranicus* and *P. patimari*. The circle area is proportional to the frequency of each haplotype. Each color indicates a sampling site (as Figure 1), each soft line connecting neighboring haplotypes represents a single mutational step, and the lines with hatch marks/numbers indicate multiple mutational steps between sampled haplotypes. Small black circles correspond to missing/hypothetical haplotypes. (b) Revised distributional ranges for *P. iranicus* and *P. patimari*, and spatial distributions of the main *P. patimari* lineages (i.e., Hg1, Hg2, and Hg3). The left star and the right star on the map represent the type localities of *P. iranicus* (upper Sefidroud sub‐basin, Tutkabon Stream, 36°50.756′N, 49°35.021′E) and *P. patimari* (Kheirud, 36°36'46.0"N 51°34'03.0"E), respectively.

GTR+I+G was determined as the best fitting nucleotide substitution model for the COI dataset (108 sequences, 648 bp length). The nucleotide substitution pattern based on Xia et al. (2003) method showed that the sequences have not reached substitution saturation and are suitable for phylogenetic analysis (*Iss* < *Iss.cS* and *Iss* < *Iss.cA*; Table S3). In total, the resulting ML tree included COI sequences for 95 samples from the SCB initially classified as *Ponticola iranicus* (*N* = 77) and *P. patimari* (*N* = 18) based on the geographical origin of the samples and current literature (Table 1; Figure 1), combined with 13 archived sequences (Table S1) from another twelve *Ponticola* species from the Ponto‐Caspian basin (Figure 3). The deepest split in the tree is between *P. ratan* and the remainder of *Ponticola* species with two fully resolved clades. One clade comprising *P. syrman* and all individuals from the SCB initially tagged with the names *P. iranicus* and *P. patimari*, and a second clade comprising the remainder of the species. Within the clade corresponding to the *P. syrman group*, *P. syrman* is sister to the clade containing all the south Caspian individuals initially assigned to *P. iranicus* and *P. patimari*. This *P. iranicus* + *P. patimari* clade divides into two subclades with a 2.32% average K2P divergence (Figures 3 and 4). The smaller subclade comprises all individuals collected from Sefidroud at Imamzadehashem (IM), close to the type locality of *P. iranicus* [upper Sefidroud sub‐basin, Tutkabon Stream, 36°50.756′N, 049°35.021′E; Vasil'eva et al., 2015]. The larger subclade with three haplogroups (i.e., Hg1, Hg2, and Hg3) includes the reminder of specimens which were initially assigned to *P. iranicus* or *P. patimari*. Hg1 contains all the collected specimens from the eastern localities in the central Mazandaran sub‐basin (Figure 4), where *P. patimari* is also described (i.e., TO, CH, NO, and KH, with the latter as the type locality of *P. patimari* Eagderi et al., 2020). Hg2 divides into two geographically oriented groups of haplotypes from PO (west Mazandaran sub‐basin) and Sefidroud at KY (Sefidroud sub‐basin). Hg3 divides into three clusters: (i) one cluster comprising all the haplotypes from SH (Talesh sub‐basin); (ii) a second cluster shared by SI (Anzali sub‐basin), KA (Talesh sub‐basin) and KY; and (iii) a third cluster comprising the remaining haplotypes from SI and one haplotype from PO.

The SP and ASAP (the partition with the highest ASAP‐score) species delimitation analyses found support for two putative species in the freshwater clade of the *P. syrman group*: one species included all specimens from Sefidroud at IM, and another species including the rest of the distribution (Hg1 + Hg2 + Hg3). The partition with the second best ASAP‐score found support for three putative species corresponding to (i) all specimens from Sefidroud at IM, (ii) Hg1, and (iii) Hg2 + Hg3. In addition, if the K2P distance species threshold value (≥2%; Ward, 2009) is accepted, IM, and Hg1 + Hg2 + Hg3 would represent two separate species (Table 2).

**TABLE 2:** Average K2P genetic distances (%, below the diagonal) between the freshwater lineages of the *P. syrman group* based on mitochondrial COI (see Figure 3).

	IM	Hg1	Hg2	Hg3
IM		0.59	0.55	0.51
Hg1	2.78		0.49	0.50
Hg2	2.33	1.89		0.31
Hg3	2.11	1.91	1.19	

Abbreviation: SD, standard deviation (above the diagonal).

A haplotype network of the south Caspian samples is shown in Figure 4a. Similar to the ML tree, two main subclades separated by 11 fixed mutational steps are evident. The 14 samples from Sefidroud at IM corresponding to the smaller subclade on Figure 3 (labeled as *P. iranicus*) defined four closely related private haplotypes. On the other hand, sequence analysis of 81 samples (from nine localities) of the larger sub‐clade on Figure 3 (labeled as *P. patimari*), detected 35 variable nucleotide sites (5 singleton variable and 30 parsimony informative sites), which defined a total of 35 haplotypes (H1–H35). H1–H7, constituent of Hg1, were closely related and separated from one another mostly by one and rarely two mutational steps: H1 private to KH, H2 private to TO, H3 shared between CH and NO, and H4–H7 private to CH. H21–H34, deviating from each other by one or two mutational steps formed Hg2 with two clusters: one cluster included H21–H27 and private to PO, the other cluster comprised H30–H33 and private to KY. The rest of haplotypes (H8–H20, H29, and H35) comprised Hg3 with numerous alternative mutational pathways (i.e., reticulation) among haplotypes: H8–H15 private to SI, H16 shared between SI and PO, H29 private to KY, H35 private to KA, and H17–H20 private to SH. Hg1 and Hg2 are deviated from Hg3 by six and two mutational steps, respectively (Figure 4a).

Based on this phylogenetic, species delimitation and phylogeographic framework, and based on the type localities of *P. iranicus* and *P. patimari*, we revise the presently known distributional ranges of the two species (Figure 4b), which is a prerequisite for the following analyses. *Ponticola iranicus* is distributed in the upper Sefidroud sub‐basin including IM, while the rest of distribution (i.e., KA, SH, SI, KY, PO, TO, CH, NO, and KH) mitochondrially belongs to *P. patimari*: Hg1 is distributed in the eastern part, Hg2 is distributed in the central part, and Hg3 with a western‐central distribution.

**FIGURE 5:**
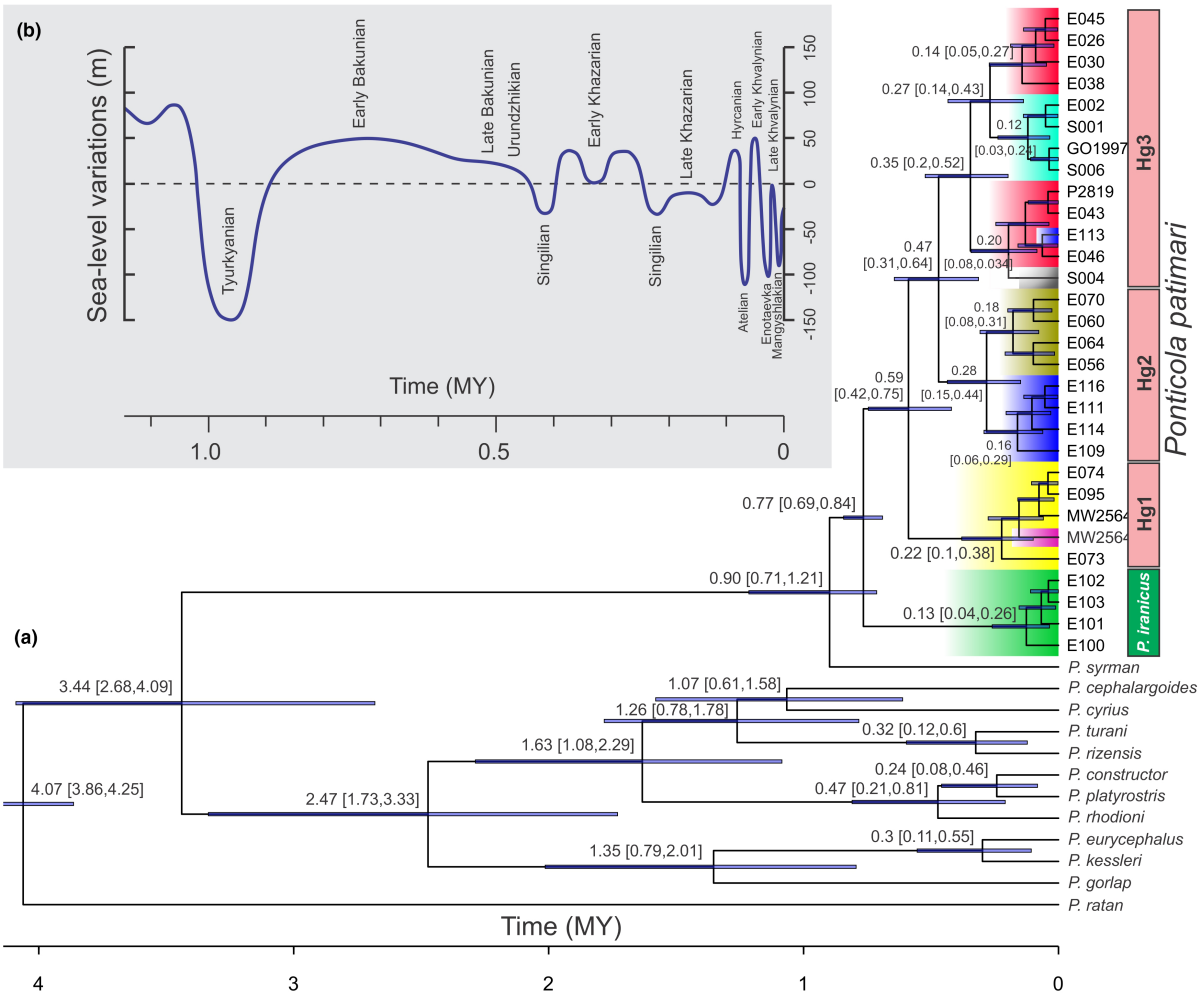
(a) Time‐calibrated phylogeny of *Ponticola*, based on a reduced COI dataset. The phylogeny is calibrated with a legacy date of 4.07 Mya at the base of *Ponticola* (Neilson & Stepien, 2009a, 2009b), and 0.9 Mya at the base of the *P. syrman group* (Zarei, Esmaeili, Abbasi, et al., 2021a; Zarei, Esmaeili, Schliewen, et al., 2021b). Error bars indicate 95% highest posterior density (HPD). (b) Schematic reconstruction of the Caspian Sea water‐level curve during the Pleistocene to Holocene (after Krijgsman et al., 2019).

Based on the calibrated time tree (Figure 5a,b), the *P. iranicus* + *P. patimari* clade diverged from *P. syrman* at 0.90 Mya (95% highest posterior density, hereafter HPD: 0.71–1.21 Mya) during the Tyurkyanian stage. Subsequent cladogenetic events dating to early Middle Pleistocene (Early Bakunian) at 0.77 Mya (95% HPD: 0.69–0.84 Mya) isolated the *P. iranicus* clade sampled at IM from *P. patimari* (Hg1 + Hg2 + Hg3). Later cladogenetic events within *P. patimari* between Hg1 and Hg2 + Hg3 dated to the temporal distance between the Early and Late Bakunian stages (0.59 Mya, 95% HPD: 0.42–0.75 Mya), whereas the divergence between Hg2 and Hg3 took place during the Urundzhikian, Singilian and Early Khazarian stages (0.47 Mya, 95% HPD: 0.31–0.64 Mya). The last major divergence within Hg3 occurred at 0.35 Mya (95% HPD: 0.20–0.52 Mya) during the Early Khazarian stage. The divergence between the PO and KY haplotypes within Hg2 and between the SH and SI haplotypes within Hg3, took place between Early Khazarian and Singilian stages (0.28 Mya, 95% HPD: 0.15–0.44 Mya; and 0.27 Mya, 95% HPD: 0.14–0.43 Mya, respectively). Subsequent diversification within *P. iranicus* from IM, Hg1, and sub‐clusters of Hg2 and Hg3 date back to the Late Khazarian (Figure 5a,b).

### 3.2 Mitochondrial DNA variability in *P. iranicus* and *P. patimari*

TO, NO, KH, and KA were excluded from the locality‐level variability analysis due to insufficient sampling. *Ponticola iranicus* samples from IM belong to four haplotypes (Table 3). It showed Hd and π values of 0.571 and 0.0012, respectively. For *P. patimari* (81 samples, 35 haplotypes), overall Hd was 0.958, ranging from 0.543 for SH to 0.902 for SI (Table 3). CH, KY and PO presented Hd values of 0.593, 0.867 and 0.882, respectively. Overall π for *P. patimari* was 0.0118, ranging from 0.001 for SH to 0.0058 for KY. CH, SI and PO presented π values of 0.0025, 0.0038, and 0.0042, respectively. Lower levels of variability in SH and CH, and higher levels of variability in KY, PO, and SI are also evident in lower and higher values of k (0.67 and 1.62 vs. 3.80, 2.75 and 2.52, respectively). Hg2 (0.924) and Hg1 (0.693) showed the highest and lowest Hd values, respectively. Hg3 showed Hd value of 0.908. Hg3 (0.0059) and Hg1 (0.0026) showed the highest and lowest π values, respectively. Hg2 presented π value of 0.0046.

**TABLE 3:** Basic parameters of genetic diversity for mitochondrial COI in the studied samples/lineages of *P. iranicus* and *P. patimari* based on the revised distributional ranges in this study

Species	Pop./Lineage	*N*	*H*	*s*	Hd ± SD	π ± SD	*k* ± SD
*P. iranicus*	IM	14	4	3	0.571 ± 0.13	0.0012 ± 0.0010	0.78 ± 0.60
*P. patimari*	TO^a^	1	1	–	–	–	–
	CH	14	5	5	0.593 ± 0.14	0.0025 ± 0.0017	1.62 ± 1.02
	NO^a^	1	1	–	–	–	–
	KH^a^	2	1	–	–	–	–
	KA^a^	2	1	–	–	–	–
	SH	15	4	2	0.543 ± 0.13	0.0010 ± 0.0009	0.67 ± 0.54
	SI	18	9	7	0.902 ± 0.04	0.0038 ± 0.0024	2.52 ± 1.42
	KY	10	6	10	0.867 ± 0.09	0.0058 ± 0.0036	3.80 ± 2.09
	PO	18	9	13	0.882 ± 0.05	0.0042 ± 0.0026	2.75 ± 1.53
	Hg1	18	7	6	0.693 ± 0.11	0.0026 ± 0.0018	1.74 ± 1.06
	Hg2	24	13	14	0.924 ± 0.03	0.0046 ± 0.0028	3.05 ± 1.65
	Hg3	39	15	14	0.908 ± 0.03	0.0059 ± 0.0034	3.88 ± 1.98
	Total	81	35	35	0.958 ± 0.01	0.0118 ± 0.0062	7.79 ± 3.66

Abbreviations: *H*, number of haplotypes; Hd, haplotype diversity; *k*, average number of nucleotide differences; *N*, number of fish; *s*, number of polymorphic sites; SD, standard deviation; π, nucleotide diversity.

^a^ Due to insufficient sampling, diversity parameters were not estimated for this samples.

### 3.3 Population differentiation and genetic structure within *P. patimari*

Excluding TO, NO, KH, and KA due to small sample sizes, the range of pairwise Fst values between the *P. patimari* samples was moderate to high and statistically significant (*p* < .001), ranging from 0.365 between two geographically close samples KY/PO, to 0.913 between two geographically distant samples SH/CH (Table 4). Since the *P. patimari* samples were distributed along an east‐west axis, we expected geographic isolation to contribute to genetic affinities. Concordantly, when all *P. patimari* localities were analyzed, an overall significant positive correlation was found between geographical and genetic distance matrices (*r* = .71, *p* < .001). Furthermore, a second Mantel test without taking into account the samples with small sample size (i.e., TO, NO, KH, and KA), indicated that the positive correlation was still high and significant (*r* = .86, *p* < .001).

**TABLE 4:** Pairwise Fst values (lower left matrix) among *P. patimari* (revised distribution) samples

	SI	KY	PO	CH	SH
SI		40.09	89.41	193.63	35.51
KY	0.476*		49.32	153.68	73.80
PO	0.632*	0.365*		104.75	122.42
CH	0.819*	0.757*	0.824		227.16
SH	0.669*	0.727*	0.787*	0.913*	

*Note*: Geographic distances between samples (in km) are presented above the diagonal.

* *p* < .001.

AMOVA for classification of all *P. patimari* localities based on three geographic regions (i.e., central, western, and eastern) revealed that 53.23% of total variation was significantly correlated with differences among the geographical regions, whereas inter‐ and intra‐sample differences explained 25.56% and 21.21% of the variation, respectively (Table 5). When samples were classified according to their sub‐basins as groups (i.e., Talesh, Anzali, Sefidroud, and Mazandaran), percentage of variance (0.45%) was lower than among samples within groups (75.63%), and not significant. A second regional‐based AMOVA for the populations with large sample sizes (i.e., SH, SI, KY, PO, and CH) also indicated that a regional classification of populations (among groups variation: 49.01%) for the observed variability significantly gave a highly better fit than the one based on sub‐basin classification.

**TABLE 5:** AMOVA for *P. patimari* (revised distribution) based on geographic region and sub‐basin classification of samples

Grouping	Analysis	Source of variation	df	Sum of squares	Variance components	% of variation (*p*‐value)
Geographic regions	A1	AG	2	177.937	2.68566 Va	53.23 (.002)
		APWG	6	56.512	1.28986 Vb	25.56 (<.001)
		WP	72	77.044	1.07006 Vc	21.21 (<.001)
		Total	80	311.494	5.04558	
	A2	AG	2	154.934	2.35904 Va	49.01 (.04)
		APWG	2	41.768	1.35407 Vb	28.13 (<.001)
		WP	70	77.044	1.10063 Vc	22.86 (<.001)
		Total	74	273.747	4.81374	
Sub‐basins	A3	AG	2	100.202	0.02028 Va	0.45 (.39)
		APWG	6	134.248	3.38387 Vb	75.63 (<.001)
		WP	72	77.044	1.07006 Vc	23.92 (<.001)
		Total	80	311.494	4.28382	

*Note*: A1: western group (SI, SH, and KA), central group (KY and PO), eastern group (TO, CH, NO, and KH). A2: western group (SI and SH); central group (KY and PO), eastern group (CH). A3: Talesh sub‐basin (KA and SH), Anzali + Sefidroud sub‐basins (SI and KY), Mazandaran sub basin (PO, TO, CH, NO, and KH).

Abbreviations: AG, among groups; APWG, among populations within groups; df, degree of freedom; WP, within populations.

### 3.4 Historical demography

**FIGURE 6:**
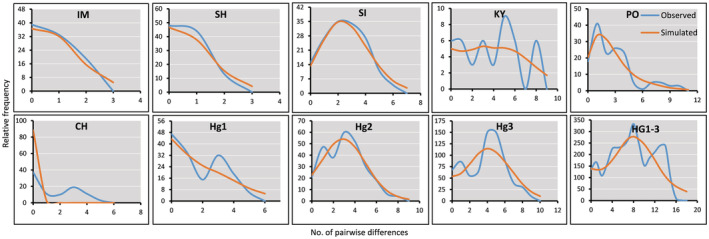
Mismatch distributions (MMDs) for *P. iranicus* (IM), and the studied samples (SH, SI, KY, PO, and CH) and lineages (Hg1, Hg2, and Hg3) of *P. patimari* (revised distributions). The observed distributions (blue lines) are compared for their goodness‐of‐fit with a Poisson distribution under a model of sudden expansion illustrated by the orange lines

TO, NO, KH, and KA were excluded from the locality‐level analyses. Estimates of Tajima's D were negative for IM (*P. iranicus*) and PO, while positive for CH, SH, SI, and KY, however, none of these values were statistically significant (*p* > .5; Table 6). Estimates of Fu's Fs and R2 were negative (*p* > .02) and low (*p* > .05) for the studied localities, respectively, indicating an excess of rare mutations compared with the expectation under a neutral model of evolution; however, these deviations from neutrality were not statistically significant. Of the three neutrality tests included, only Fu's Fs significantly (*p* < .02) supported a recent demographic expansion for the pooled dataset of *P. patimari* (Hg1 + Hg2 + Hg3). In haplogroup‐level analysis, estimates of Tajima's D were negative for Hg1 and Hg2, while positive for Hg3, however, none of these values were statistically significant (*p* > .5). Estimates of Fu's Fs were negative for the haplogroups, but only significant for Hg2 (*p* < .02). Estimates of R2 were non‐significantly low for the studied lineages (*p* > .05). MMD when applied to *P. iranicus* from IM (Figure 6; Table 6), was unimodal and unable to reject a model of sudden expansion (*p* (sim ≥ obs) > .05). For two of the *P. patimari* localities, SH and SI, MMDs were unimodal and unable to reject the model of sudden expansion (*p* (sim ≥ obs) > .05). MMD for the pooled data set of *P. patimari* (Hg1 + Hg2 + Hg3) revealed a multimodal mismatch distribution (Figure 6). In haplogroup‐level analysis, the non‐unimodal MMDs also rejected the model of sudden expansion for the *P. patimari* haplogroups. Accordingly, because of discrepancies between the results of neutrality tests and MMDs, we were not able to estimate the time elapsed since the beginning of demographic expansion for any of the studied localities, haplogroups, and species.

**TABLE 6:** Demographic tests for mitochondrial COI in the studied samples/lineages of *P. iranicus* and *P. patimari* based on the revised distributional ranges in this study

Species	Sample/Lineage	*N*	Tajima's D	Fu's Fs	R2	SSD (*p*‐value)	Hri (*p*‐value)
*P. iranicus*	IM	14	−0.529	−0.959	0.142	0.002 (.75)	0.072 (.88)
*P. patimari*	CH	14	0.095	−0.449	0.168	0.438 (.001)	0.108 (1.00)
	SH	15	0.221	−1.223	0.167	0.004 (.80)	0.104 (.64)
	SI	18	0.813	−2.746	0.180	0.001 (.92)	0.025 (.91)
	KY	10	0.332	−0.256	0.175	0.026 (.55)	0.089 (.51)
	PO	18	−1.021	−2.425	0.120	0.011 (.55)	0.053 (.51)
	Hg1	18	−0.011	−1.869	0.137	0.014 (.67)	0.051 (.85)
	Hg2	24	−0.649	−5.334*	0.099	0.005 (.67)	0.028 (.74)
	Hg3	39	0.540	−3.525	0.138	0.012 (.28)	0.028 (.43)
	Total	81	0.332	−10.816*	0.109	0.005 (.57)	0.006 (.88)

Abbreviations: Hri, Harpending's raggedness index; *N*, number of fish; R2, Ramos‐Onsins and Rozas's R2; SSD, sum of squared deviations.

* *p* < .01.

### 3.5 Lateral line system

#### 3.5.1 *Ponticola patimari* from KH (haplogroup Hg1), the species type locality

**FIGURE 7:**
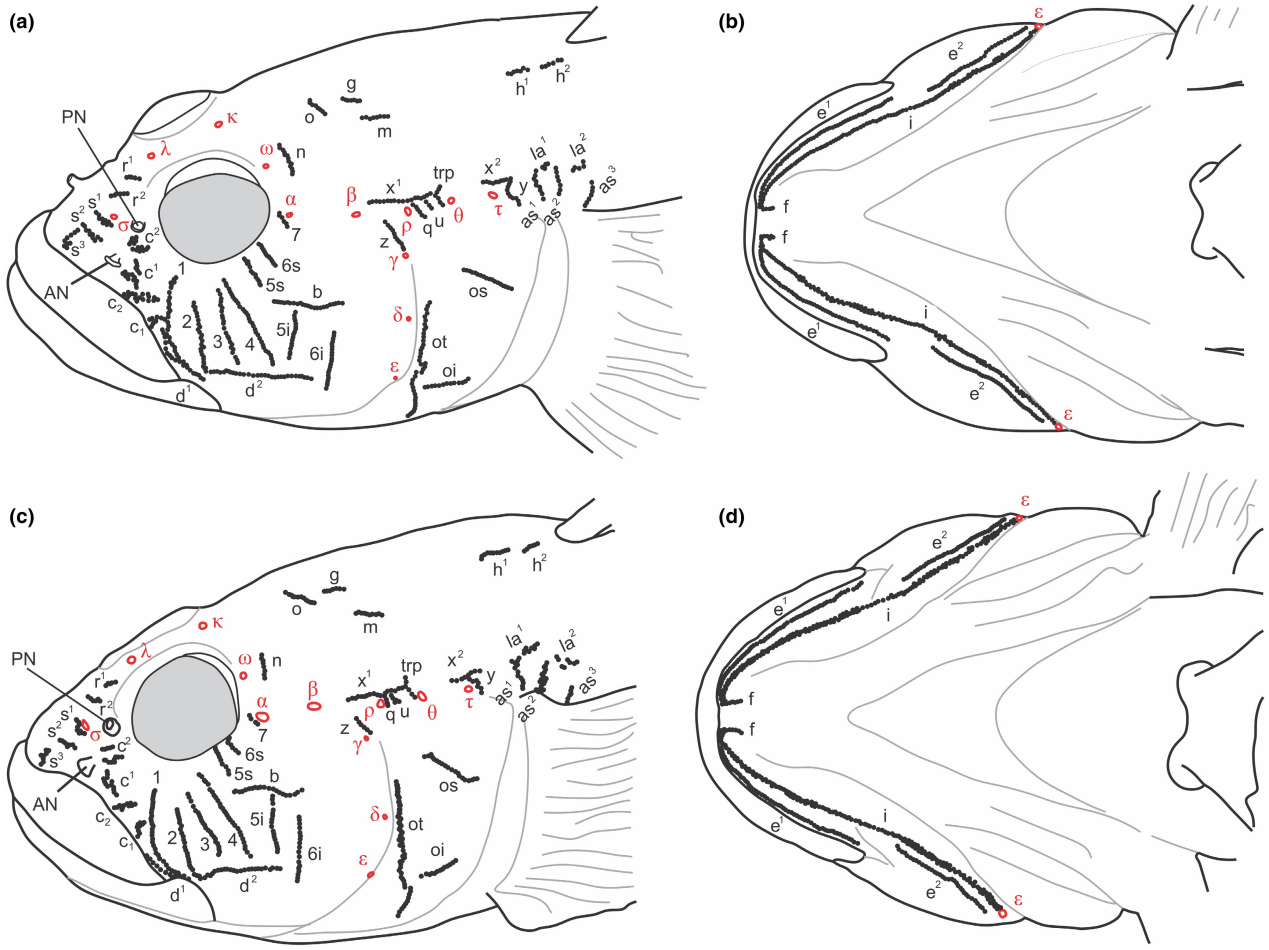
Head sensory papillae pattern of lateral line system and canal pores (Greek lettering) of *P. patimari* from KH (a, b; 91.46 mm SL, ZM‐CBSU S088‐4; Hg1) and *P. iranicus* from Sefidroud at IM (c, d; 80.54 mm SL, ZM‐CBSU S087‐1). Terminology in the text

***Cephalic canals* (Figure** **7** **a).** Head with AOC, POC, and PC canals and pores. AOC with a single, unified interorbital section and carrying 12 pores: a pair of posterior nasal pores *σ*, single interorbital pores λ and κ, and paired *ω*, *α*, *β*, and *ρ*; pores *σ* and *ρ* terminal, pore *λ* directly on the canal, pores *κ* and *ω* behind the canal, and pores *α* and *β* lateral of the canal. POC paired, each with two pores: *θ* and *τ*. PC paired, each side with three pores: *γ*, *δ*, and *ε*.

***Head sensory papillae* (Figure** **7** **a,b).** *Preorbital*: median series in five rows: rows *r* ^*1*^ and *r* ^*2*^ as oblique single rows medial to posterior nostril, extending over the canal section between pores *λ* and *σ*; rows *s* ^*1*^ and *s* ^*2*^, as transverse rows anterior to pore *σ*, partially doubled; row *s* ^*3*^ anteriorly as cluster anterior and lateral of row *s* ^*2*^, reaching near to upper lip; lateral series in four rows; row *c* ^*2*^ between the anterior and posterior nostrils as two clusters, with lower section often longer than upper; row *c* ^*1*^ as two transversal row lateral of anterior nostril and dorsal of row *c*_*2*_; rows *c*_*2*_ and *c*_*1*_ longitudinal and oblique, respectively, partially doubled, above posterior upper lip; row *c*_*2*_ ventral to row *c* ^*1*^, row *c*_*1*_ ventral to row *c*_*2*_ and dorsal to row *d* ^*1*^, both posteriorly close to suborbital row *1*. *Suborbital*: seven transversal (*1*–*7*) and two longitudinal (*b*, *d*) rows on cheek, all as single row of papillae, not doubled or tripled in width, rows *1*–*4* before longitudinal row *b*, long, ventrally extending to level of row *d*, dorsally reaching close to eye except row *2* and sometimes row *3* ending distantly from eye; rows *1* and *2* above and anterior to rear edge of jaws, row *3* right above or slightly behind the jaws angle; row *5* and *6* divided by row *b* in short superior (*5s*, *6s*) and longer inferior (*5i*, *6i*) sections; row *5i* ending above longitudinal row *d*, row *6i* passing behind row *d*, ending slightly below its level; row *5i* and *6i* not confluent with each other; row *7* short (3–6 neuromasts), immediately anterior of pore *α*; row *b* anteriorly reaching to below posterior end of pupil; row *d* long, not reaching row *6i* posteriorly, often distinctly divided and separable in two slightly overlapping parts, the anterior supralabial row *d* ^*1*^ oblique, following the border of the upper lip and reaching below the anterior origin of row *d* ^*2*^, the posterior row *d* ^*2*^ longitudinal on cheek; row *d* ^*1*^ anteriorly passing row *1*. *Preoperculo‐mandibular*: three rows, *e*, *i* and *f*; external row *e* distinctly divided at articulation of lower jaws in anterior mandibular (*e* ^*1*^) and posterior preopercular (*e* ^*2*^) sections; anterior and posterior sections of internal row *i* continuous, their separation at articulation of lower jaws is indistinct; row *i* doubled in part and anteriorly continuous with the mental row *f*, row *f* longitudinal. *Oculoscapular*: eight transversal (*z*, *q*, *u*, *trp*, *y*, *as* ^*1*^–*as* ^*3*^) and four longitudinal (*x* ^*1*^, *x* ^*2*^, *la* ^*1*^–*la* ^*2*^) rows including the axillary series; row *x* ^*1*^ long, parallel to above AOC, reaching to *trp*; row *x* ^*2*^ posterior to rows *x* ^*1*^ and *trp*, above posterior third of opercle and above pore *τ*; row *z* upward from the PC dorsal end, ventrally starting close to, but not exceeding pore *γ*; row *y* behind pore *τ* and below row *x* ^*2*^; three short transverse rows in oculoscapular groove between pores *ρ* and *θ*: row *q* anteriormost, close to pore *ρ*, extending ventrally of the oculoscapular groove and sometimes with one or two papillae dorsal of pore *ρ*; posteriormost row *trp* close to pore *θ*, extending dorsally and passing upwards the level of row *x* ^*1*^; between rows *q* and *trp* a third transverse row named here row *u* considering its position despite row being transverse; transversal axillary rows *as* ^*1*^–*as* ^*3*^ long; longitudinal axillary series represented by two rows (*la* ^*1*^, *la* ^*2*^). *Opercular*: three rows, transversal row *ot*, sometimes divided in two parts, and two longitudinal rows, upper *os* and lower *oi*). *Anterior‐dorsal (occipital)*: five rows, two transversal rows (*n*, *o*) and three longitudinal rows (*g*, *m*, *h*); *n* behind pore *ω* of AOC; rows *o* widely separated; row *g* posterior of row *o*, not reaching row *o* anteriorly; row *m* almost parallel to row *g*, behind and below it; row *h* anterior to origin of first dorsal fin, divided in two sections (*h* ^*1*^, *h* ^*2*^).

#### 3.5.2 *Ponticola iranicus* from IM, near to the species type locality, and *P. patimari* Hg2–Hg3

**FIGURE 8:**
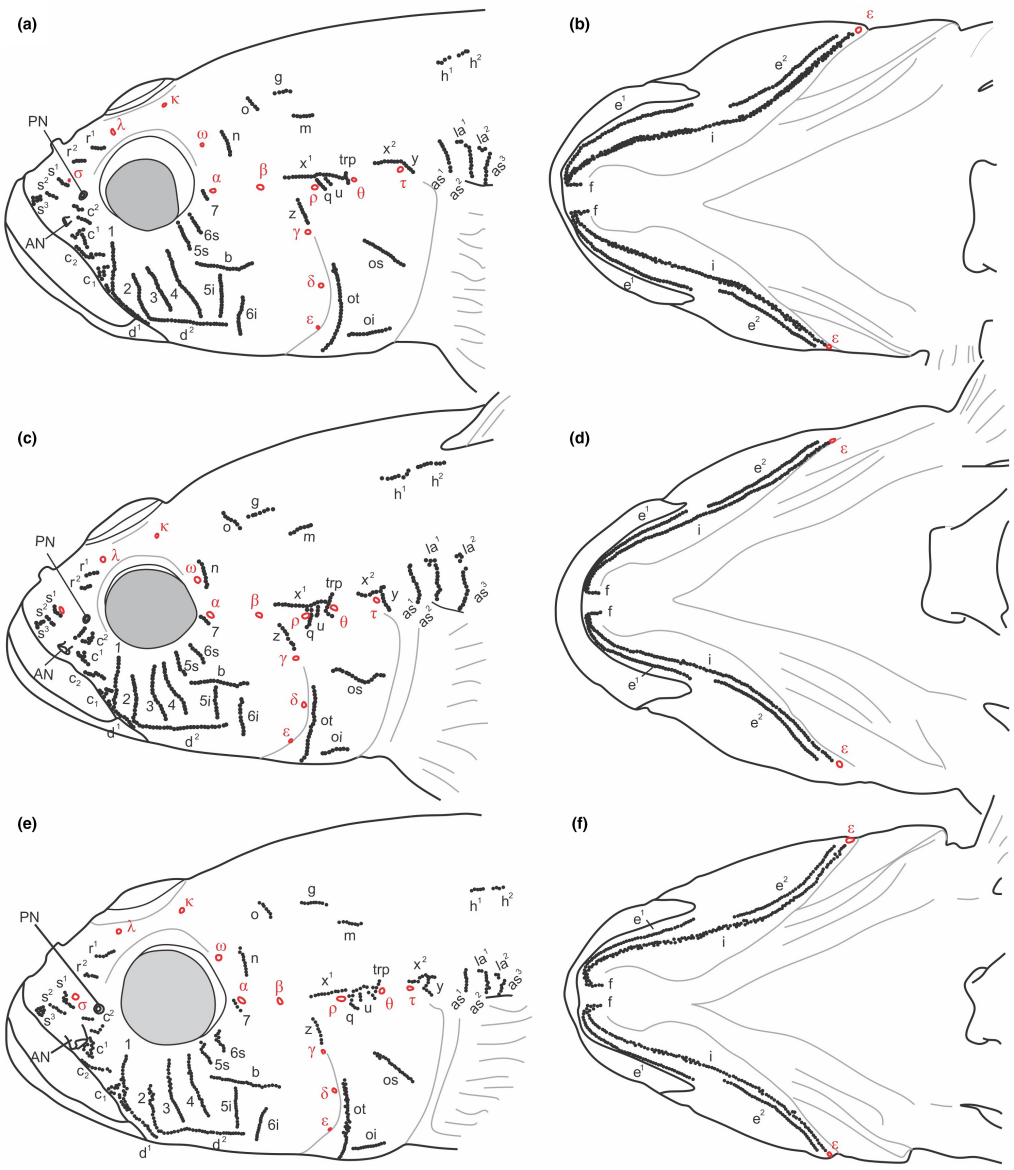
Head sensory papillae pattern of lateral line system and canal pores (Greek lettering) of *P. patimari* from PO (a, b; 75.57 mm SL, male, ZM‐CBSU PL3; Hg2), KA (c, d; 71.21 mm SL, male, ZM‐CBSU S044/1; Hg3), and SI (e, f; 66.88 mm SL, male, ZM‐CBSU E035; Hg3). Terminology in the text

Head lateral‐line patterns in *P. iranicus* (Figure 7c,d) from IM, and in other *P. patimari* haplogroups (i.e., Hg2 and Hg3; Figure 8) are quite similar to that of *P. patimari* from the species type locality (i.e., KH: Hg1). AOC, POC, and PC canals present, with pores *σ*, *λ*, *κ*, *ω*, *α*, *β*, and *ρ*; *θ* and *τ*; and *γ*, *δ*, and *ε*, respectively. Suborbital infraorbital neuromast organs in seven transverse rows, four rows (*1*–*4*) before and three rows (*5s*, *6s* and *7*) above and two rows (*5i*, *6i*) below row *b* and row *a* absent, row *7* consists of a few to several papillae descending from before anterior oculoscapular pore *α*, rows *5i* and *6i* separated, with row *5i* well behind anterior end of row *b* and row *6i* close to posterior end of row *b*; dorsal rows *o* separated in dorsal midline; oculoscapular row *z* ends near to pore *γ*; row *x* ^*1*^ ending anteriorly behind pore *β*.

### 3.6 General morphology of sagittae otolith inner face

**FIGURE 9:**
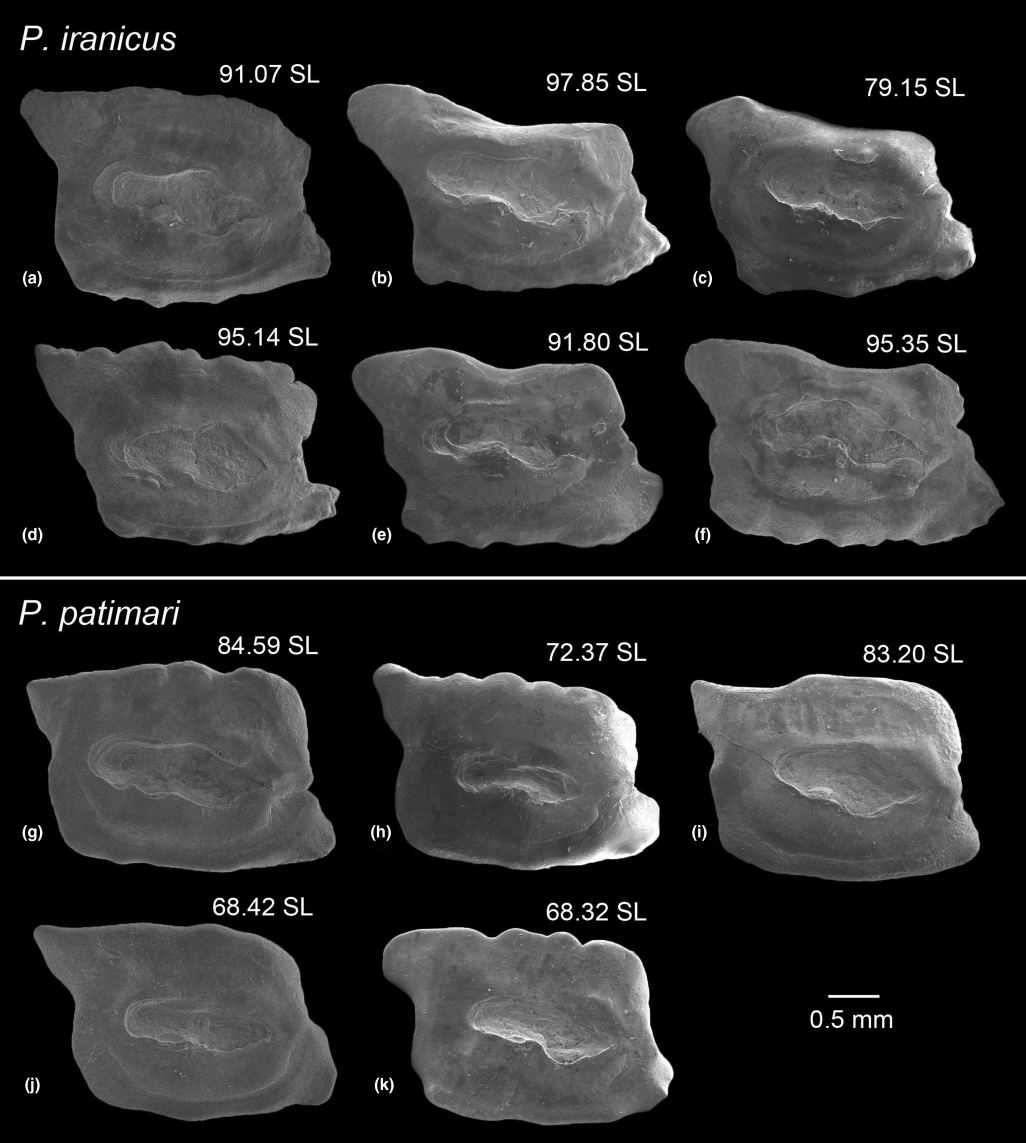
Otoliths (mesial view) of *P. iranicus* (a–f: Sefidroud at IM, ZMCBSU 37–40, 141, 144) and *P. patimari* (g–k: CH, ZMCBSU P1–P4, P6). Standard length (SL) is presented in mm

The otoliths of *P. iranicus* from IM and *P. patimari* from NO (samples near to the species type localities) have parallelogram shapes, with marked preventral and posterodorsal projections (Figure 9). The OL/OH ratios are similar. In *P. iranicus* from IM, dorsal rim straight or posteriorly elevated, sometimes with a shallow broad concavity in the middle, smooth or undulated (vs. straight, smooth or undulated); predorsal angle obtuse or nearly orthogonal (vs. usually orthogonal or sometimes obtuse); ventral rim horizontal, usually undulated (vs. horizontal, usually smooth); posteroventral angle obtuse or orthogonal (vs. usually orthogonal). Posterodorsal projection in specimens from both localities, long and broad, pointed or blunt, bent outwards, and usually facing upwards (dorsally). Anterior rim usually without incision, sometimes slightly incised at the level of ostium, usually smooth or rarely slightly undulated, inclined at 68.06–77.83° (β) and 66.10–78.87°, respectively. Posterior rim almost parallel to the anterior rim, inclined at 103.67–110.92° (γ) and 98.53–109.54°, respectively, usually without incision below the projection. Angle of preventral to posterodorsal traverse, 20.90–28.61° (δ) and 22.17–27.58°, respectively. Preventral projection moderately long, pointed or blunt. Sulcus centrally positioned, sole‐shaped, long and wide, anteriorly inclined at 11.02–16.40° (α) and 7.81–13.78°, respectively, deep with moderately developed ostial lobe. Subcaudal iugum distinct or indistinct. Ventral furrow running with a moderate or close distance to ventral rim, curved upwards anteriorly to the level of the ostial apex and turning upwards posteriorly to the level of the caudal tip or slightly behind it. Dorsal depression distinct or indistinct.

### 3.7 Differences in classical otolith variables and shape descriptors

Table 7 shows the result of calculated otolith variables, inclination angles, classical shape descriptors, mean values and standard deviations for *P. iranicus* from IM (6 specimens) and *P. patimari* from NO (5 specimens). None of the variables and inclination angles were found to be useful for separation between the two species (ANOVA, *p* > .05, Tukey's HSD post‐hoc test). The otoliths of *P. iranicus* from IM and *P. patimari* from NO were only slightly different in their rectangularity index (REx; 0.628–0.729 vs. 0.732–0.781).

**TABLE 7:** Calculated otolith inclination angles, morphometric variables, and shape indices for *P. iranicus* from IM and *P. patimari* from NO

	*P. iranicus* from IM (*N* = 6)				*P. patimari* from NO (*N* = 5)			
	Min.	Max.	Mean	SD	Min.	Max.	Mean	SD
α	11.017	16.397	13.991	2.149	7.808	13.780	10.405	2.772
δ	20.898	28.610	25.412	3.204	22.166	27.582	25.535	2.255
γ	103.671	110.925	107.556	3.314	98.531	109.537	104.522	4.392
β	66.103	78.871	73.548	4.784	68.062	77.829	74.519	3.811
OL/OH	1.363	1.600	1.475	0.089	1.367	1.581	1.462	0.079
OP/OL	2.947	3.166	3.024	0.087	2.879	3.174	3.044	0.111
OP/OH	4.179	4.745	4.455	0.208	4.337	4.552	4.445	0.079
SuA/OA	0.116	0.158	0.134	0.015	0.079	0.146	0.121	0.028
SuP/OP	0.377	0.445	0.422	0.026	0.350	0.444	0.402	0.034
SuP/SuTipV	4.525	5.448	5.070	0.369	4.180	5.145	4.598	0.353
SuP/SuEndV	3.160	4.251	3.825	0.399	3.455	4.303	3.825	0.393
SuL/OL	0.479	0.550	0.519	0.024	0.442	0.562	0.503	0.045
SuL/OH	0.689	0.832	0.765	0.062	0.604	0.829	0.736	0.081
SuL/SuH	2.109	2.911	2.408	0.332	2.103	2.561	2.363	0.203
SuL/SuTipV	1.883	2.211	2.059	0.132	1.663	2.143	1.893	0.175
SuL/SuEndV	1.298	1.705	1.554	0.159	1.374	1.793	1.575	0.181
SuL/OP	0.151	0.181	0.172	0.011	0.139	0.185	0.165	0.016
SuL/SuP	0.401	0.416	0.406	0.006	0.398	0.417	0.411	0.008
SuH/OL	0.183	0.243	0.218	0.024	0.185	0.248	0.214	0.028
SuH/OH	0.285	0.359	0.320	0.024	0.252	0.353	0.313	0.040
SuH/SuTipV	0.647	1.027	0.872	0.150	0.694	0.873	0.804	0.081
SuH/SuEndV	0.566	0.808	0.652	0.092	0.574	0.704	0.667	0.053
SuH/OP	0.062	0.082	0.072	0.007	0.058	0.080	0.070	0.009
SuH/SuP	0.143	0.190	0.171	0.021	0.162	0.196	0.175	0.016
SuTipV/OP	0.072	0.096	0.084	0.008	0.084	0.092	0.087	0.003
SuTipV/SuEndV	0.655	0.895	0.757	0.091	0.778	0.911	0.832	0.050
SuEndV/OP	0.089	0.128	0.112	0.014	0.096	0.114	0.105	0.008
OL2/CL	1.419	1.674	1.494	0.098	1.451	1.820	1.606	0.134
ROx	0.541	0.658	0.606	0.044	0.590	0.699	0.660	0.042
REx	0.628	0.729	0.700	0.038	0.732	0.781	0.756	0.022
ELx	0.154	0.231	0.191	0.029	0.155	0.225	0.187	0.026

Abbreviation: *N*, number of fish; SD, standard deviation.

### 3.8 Otolith outline differences

A total of 213 specimens belonging to six samples (i.e., IM, CH, KY, PO, SH, and SI) were analyzed for their left sagittal otolith outline. According to the ANOVA‐like permutation test, the lengths of the three major radii were significantly (*p* < .01) different between all the *P. iranicus* (i.e., IM) and *P. patimari* (i.e., CH, KY, PO, SH, and SI) samples (Table 8).

**TABLE 8:** ANOVA‐like permutation test of the otolith shape between samples (1000 permutations)

Comparison	df	SS	*F*‐value	*p*‐value
IM vs. KY	1	4.64	14.42	.001
IM vs. PO	1	1.32	2.14	.001
IM vs. SH	1	13.43	11.87	.001
IM vs. SI	1	6.39	16.77	.001
CH vs. IM	1	14.41	25.22	.001
CH vs. KY	1	7.80	12.74	.001
CH vs. PO	1	5.46	11.30	.001
CH vs. SH	1	11.91	13.03	.001
CH vs. SI	1	11.4	26.67	.001
KY vs. PO	1	9.12	9.36	.001
KY vs. SH	1	0.18	7.60	.001
KY vs. SI	1	1.34	2.66	.001
PO vs. SH	1	2.82	4.18	.001
PO vs. SI	1	5.98	10.39	.001
SH vs. SI	1	12.46	12.54	.001

*Note*: *p* < .05 shows significant effect.

Abbreviations: df, degree of freedom; SS, sum of squares.

**FIGURE 10:**
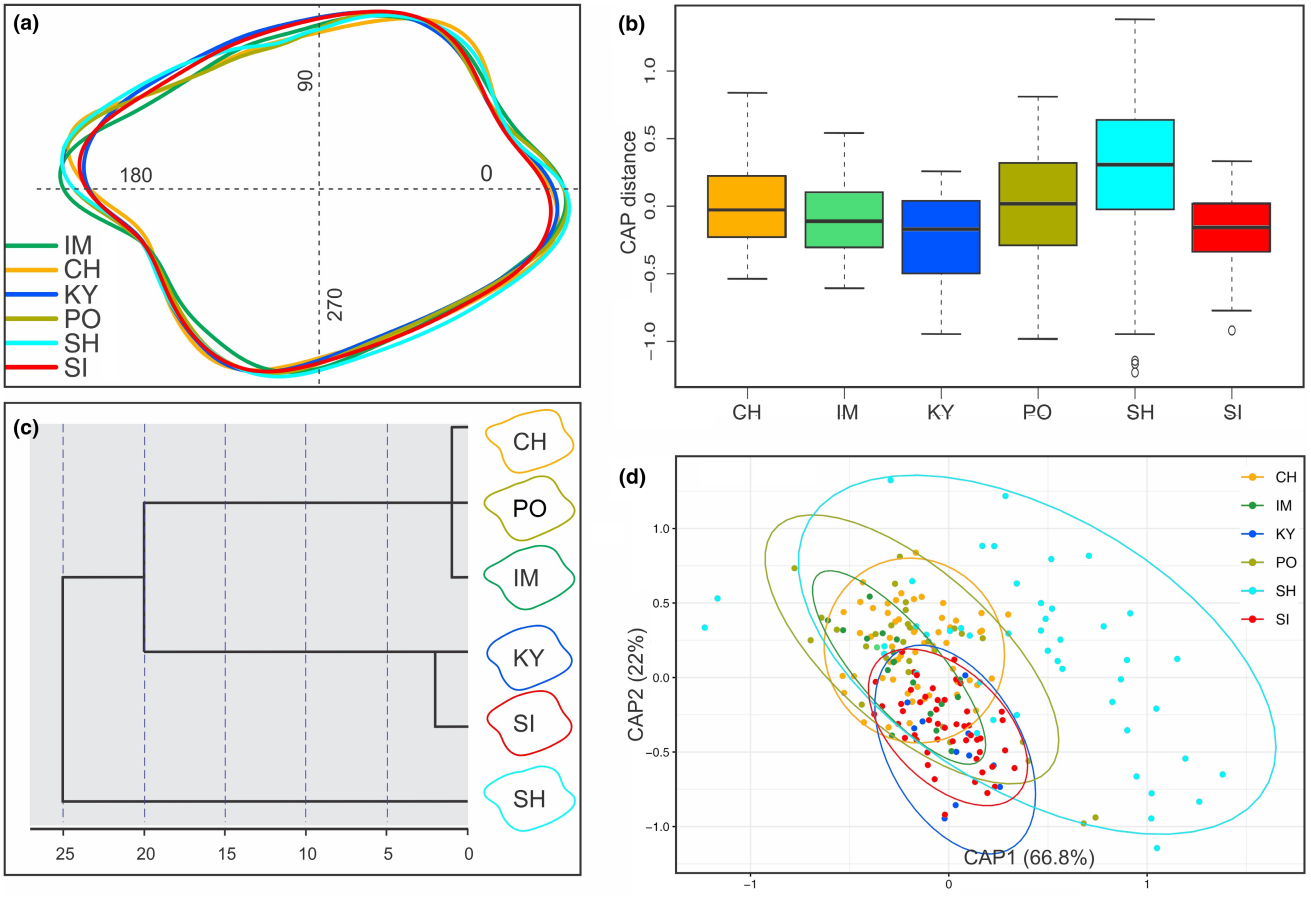
(a) Mean otolith shapes based on wavelet reconstruction for the studied samples. The numbers 0, 90, 180 and 270 indicate angle in degrees (°) on the outline. (b) Boxplots of canonical score distances with respect to variation among samples based on the wavelet coefficients. (c) Hierarchical cluster analysis based on otolith outline data (CAP1, CAP2) and using the Euclidean distance showing the phenotypic relations among samples (mean otolith outlines are depicted on the right). (d) Canonical scores on discriminating axes 1 (CAP1) and 2 (CAP2) for the studied samples using wavelet methodology. Individual data points are indicated by the colored circles.

The mean otolith outline based on the wavelet coefficients differed among the studied samples mainly at the posteroventral, posterodorsal and preventral regions (Figure 10a). At the preventral projection (350–10°), SH shows the outermost otolith outline (i.e., longest projection) followed by PO and IM, moving towards the otolith centroid in the innermost part is SI (i.e., shortest projection). At the level of anterior concavity (20–30°), IM and SI show the outermost and innermost outlines, respectively. At the predorsal angle (40–55°), CH shows the most projected outline, forming almost a right angle. Having a longer preventral projection and a more projected predorsal angle, SH also shows a pronounced concavity on the anterior rim. Running posteriorwardly to the level of 90°, CH shows the most depressed outline. KY and PO showed the outermost and innermost otolith outlines at the middle of dorsal rim, respectively. From mid‐dorsal running backwards to the posterodorsal projection, they are replaced by SH and IM, respectively. At the posterodorsal projection, IM followed by SH show the farthest otolith outlines from the centroid (i.e., longest projections), and moving inward, KY followed by SI are in the innermost parts (i.e., shortest projections). Below the projection to the level of posterior incision (180–200°), IM and CH show the outermost and innermost outlines, respectively. At the posteroventral angle (205–240°), IM shows the innermost otolith outline, and below the angle running towards the preventral projection, SH is in the outermost part.

Boxplots of canonical score distances with respect to variation among samples based on the wavelet coefficient indicate shape differences between SH and the other samples, but no significant differences observed among all of the other samples (Figure 10b). A cluster analysis dendogram based on CAP1 and CAP2 and using the Euclidean distance (Table S4) showed two main clusters, SH in one, and the other samples in the other (Figure 10c). The larger cluster is divided into two sub‐clusters: one including KY and SI, and the other including CH, PO, and IM. The first two discriminating axes of the CAP analysis based on the wavelet coefficients explained 88.8% (CAP1 = 66.8%, CAP2 = 22%) of the variation between samples, showing a clear differences between SH and the others (Figure 10d). The overall classification success with a leave‐one‐out cross‐validation estimation was 43.7%, the highest classification success was achieved for SH (71.7%), followed by KY (54.5%) and IM (47.4%; Table 9). The Mantel test confirmed that there was no correlation between the otolith outline variation and the geographical distances among samples (*r* = .37, *p* > .05). The levels of otolith outline resemblance between samples were somewhat dependent on genetic distance (*r* = .54, *p* = .019).

**TABLE 9:** Classification values based on Linear Discriminant Analysis of the standardized wavelet coefficients of the samples

Sample	CH	PO	KY	SI	IM	SH	Total
CH	**40.0**	11.7	3.3	5.0	28.3	11.7	100
PO	48.3	**3.4**	13.8	6.9	24.1	3.4	100
KY	0.0	9.1	**54.5**	27.3	9.1	0.0	100
SI	2.1	6.3	37.5	**39.6**	14.6	0.0	100
IM	15.8	10.5	10.5	15.8	**47.4**	0.0	100
SH	8.7	2.2	4.3	6.5	10.9	**67.4**	100

*Note*: The numbers in rows are percentages that denote the classification into the sampling areas given in columns (correctly classified samples are in bold). Overall classification (cross‐validated): 43.7%.

### 3.9 Meristic and morphometric differences

To examine the degree of similarity among the three main lineages (i.e., *P. iranicus*, *P. patimari* Hg1, and *P. patimari* Hg2 + Hg3), 41 morphometric as well as eight meristic variables from 107 specimens were analyzed. The range, mean and standard deviation of each meristic and standardized morphometric measurement for the three lineages are shown in Tables S5 and S6, respectively. D1 was excluded since it was constant. One‐way ANOVA showed significant differences (*p* < .01) in mean values of five meristic variables (i.e., A, C, PD, TR, LL) between the three lineages. Tukey's HSD post‐hoc comparisons showed significant differences between *P. iranicus* and both *P. patimari* Hg1 and *P. patimari* Hg2 + Hg3 for PD, TR and LL.

ANOVA revealed significant differences (*p* < .01) in 33 size adjusted morphometric measurements between the three lineages, of which, 27 showed a separation between *P. iranicus* and both *P. patimari* Hg1 and *P. patimari* Hg2 + Hg3 by Tukey's HSD post‐hoc comparisons, while only six variables (i.e., D1b, Ab, Vd, I, AULw, ULw) showed significant differences between *P. patimari* Hg1 and *P. patimari* Hg2 + Hg3. MANOVA confirmed a high significant lineage variability (*p* < .005) for the 33 size adjusted morphometric variables with significant differences (Table 10); these variables were used for the PCA, DFA, and cluster analysis.

**TABLE 10:** MANOVA for differences among the three main lineages (*P. iranicus*, *P. patimari* Hg1, and *P. patimari* Hg2 + Hg3) based four tests.

Effect	Value	*F*	Hypothesis df	Sig.
Pillai's Trace	0.967	7.389	80.0	.00001
Wilks' Lambda	0.022	9.412	80.0	.00001
Hotelling's Trace	14.843	11.875	80.0	.00001
Roy's Largest Root	12.401	20.461	40.0	.00001

Abbreviation: df, degree of freedom.

**FIGURE 11:**
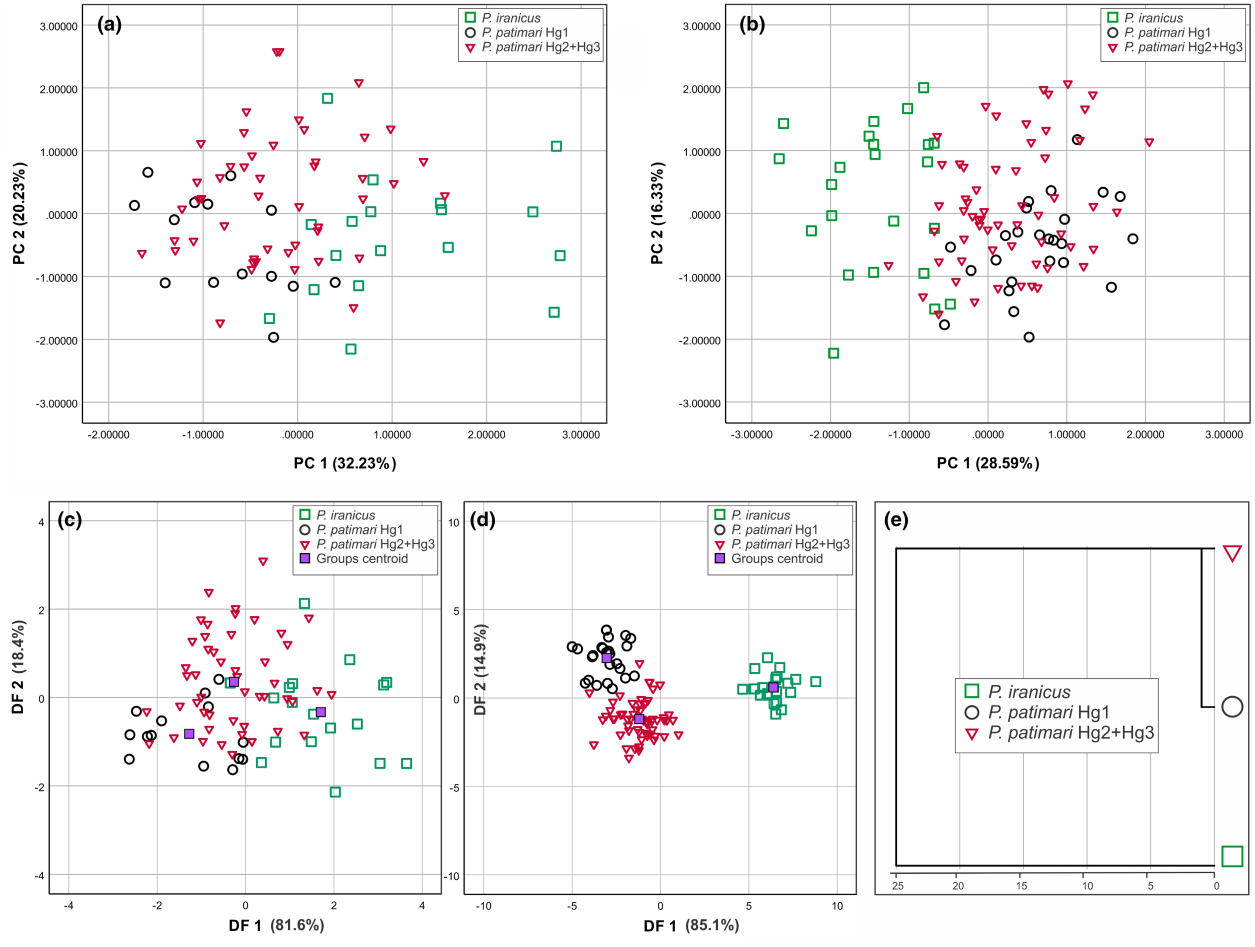
(a) Scatter plot of PC 1 vs. PC 2 for PCA using meristic variables. (b) Scatter plot of PC 1 vs. PC 2 for PCA using size‐adjusted morphometric variables. (c) Sample centroids plot of discriminant function scores using meristic variables. (d) Sample centroids plot of discriminant function axes using size‐adjusted morphometric variables. (e) Dendrogram derived from cluster analyses of meristic and size‐adjusted morphometric measurements. *Ponticola iranicus* = square; *P. patimari* Hg1 = circle; *P. patimari* Hg2 + Hg3 = triangle.

The five meristic variables, TR, PD, LL, A, P, and C were subjected to PCA and DFA. Three PCs were extracted, accounting for 69.7% of the total variation. PC 1, PC 2, and PC 3 accounted for 32.23%, 20.23%, and 17.23% of the variation, respectively. In order of importance, the most significant variables loadings on PC 1, PC 2, and PC 3 were TR, PD, LL and C and A, P, respectively. Bivariate plot of PC 1 and PC 2 scores showed a better separation of *P. iranicus* from both *P. patimari* Hg1 and *P. patimari* Hg2 + Hg3 (Figure 11a). PCA for significant morphometric variables extracted from variance‐covariance matrix revealed that PC 1, PC 2, and PC 3 accounted for 28.59%, 16.33%, and 9.57% of the variation, respectively, which gave a cumulative variation of 54.5% for the first three PCs. In order of importance, the most significant variables loading on PC 1 and PC 2 were D1III, HL, E, PO, D1I, Cl, TL, D1II, A1I, Pl, ULl, D2I, Vl, SN/D1, D2h and Lam, SN, Ulw, AULw, Hde, Hw, respectively. Bivariate plot of PC 1 and PC 2 scores supported a better separation between *P. iranicus* and both *P. patimari* Hg1 and *P. patimari* Hg2 + Hg3 (Figure 11b).

For the meristic variables, the discriminant function test using Wilks' λ statistic was significant (λ = 0.41, χ^2^ = 67.85, *p* < .0001), indicating a relatively high degree of inter lineages variance and that the means of the discriminant scores for the three lineages are different. DFA produced two discriminant functions for the meristic variables, DF 1 accounted for 81.6% and DF 2 accounted for 18.4% of among group variability. Biplot of DFA showed a separation between *P. iranicus* and both *P. patimari* Hg1 and *P. patimari* Hg2 + Hg3 (Figure 11c). DFA of size adjusted morphometric variables picked out Ad, E, SN, Pl, ULl, AULw, Hde, SN/AN, and Ulw as the main significant contributors of lineages differentiation. DF 1 accounted for 85.1% and DF 2 for 14.9% of among group variability. The DFA biplot showed morphological separation between the three lineages, especially a clear separation between *P. iranicus* and both *P. patimari* Hg1 and *P. patimari* Hg2 + Hg3 (Figure 11d).

Functions at group centroids analysis using the size adjusted variables revealed an average of 5.77 for *P. iranicus*, and −2.84 and −1.04 for *P. patimari* Hg1 and *Ponticola patimari* Hg2 + Hg3, respectively, suggesting a clear differentiation between *P. iranicus* and both *P. patimari* Hg1 and *P. patimari* Hg2 + Hg3. Classification success rates were estimated for the three lineages (Table S7). The proportion of individuals correctly classified into their original lineages was 100%, 100%, and 96% in *P. iranicus*, *P. patimari* Hg1, and *P. patimari* Hg2 + Hg3 (4% classified into *P. patimari* Hg1), respectively, indicating a high rate of correct classification of specimens. Furthermore, cluster analysis for the meristic and morphometric measurements resulted in a dendrogram with two clusters: *P. iranicus* in one, and the two *P. patimari* lineages in the other (Figure 11e).

## 4 DISCUSSION

### 4.1 Species diversity and distributions

Our molecular results based on mitochondrial COI highlighted several main features, providing support for two or three freshwater species in the *P. syrman group*. All freshwater samples of the *P. syrman group* belong to a monophyletic clade with two main subclades: (i) a smaller subclade comprising all individuals collected from the upper Sefidroud sub‐basin, where *P. iranicus* is also described (Vasil'eva et al., 2015), and (ii) a larger subclade with three geographically structured haplogroups (i.e., Hg1, Hg2, and Hg3), which includes specimens from the rest of the distribution. Hg1 contains all the specimens collected from the eastern localities, where the type locality *P. patimari* is located (Eagderi et al., 2020); Hg2 and Hg3 are sister groups with central and western‐central distributions, respectively. The two main subclades are separated by 12 mutational steps and a 2.32% K2P genetic distance in COI, while the three haplogroups within the larger subclade are separated from one another by 2–6 mutational steps and 1.19%–1.91% K2P genetic divergence, i.e., less than the conventional threshold proposed for COI in fish (i.e., 2%; Ward, 2009) used as an indicator of distinct species. Genetic divergences between the three haplogroups of the larger subclade and between Hg1 and Hg2 + Hg3 are significantly smaller than those between most species of *Ponticola* (Eagderi et al., 2020; Zarei, Esmaeili, Kovačić, et al., 2022a), except for the one between *P. kessleri* and *P. eurycephalus* (1.2%), and those between several other freshwater endemic species: *P. platyrostris* and *P. constructor* (1.2%), *P. platyrostris* and *P. rhodioni* (1.5%), *P. constructor* and *P. rhodioni* (1.8%), and *P. rizensis* and *P. turani* (1.5%). The SP and ASAP delimitation analyses indicated two distinct species, corresponding to each major subclade. The partition with the second best ASAP‐score however, found support for three hypothetical species, corresponding to: (i) the smaller subclade from upper Sefidroud, (ii) Hg1, and (iii) Hg2 + Hg3. Nevertheless, a consensus among molecular, distributional, and morphological data, i.e., integration by congruence (Padial et al., 2010), is often used as the major argument for establishing species delineation (Kekkonen & Hebert, 2014; Puillandre et al., 2021).

Eagderi et al. (2020) described *P. patimari* from KH, CH, and TO, all within the range of Hg1, and distinguished it from *P. iranicus* originally described from the upper Sefidroud sub‐basin (i.e., the range of the smaller subclade) based on a 3.0% K2P genetic distance in COI [estimated from the analysis two *P. iranicus* (one haplotype) and four *P. patimari* specimens (two haplotypes)], having six suborbital transverse papillae rows (vs. seven rows), less branched D2 rays, a longer CPd, and P reaching D2 origin (vs. not reaching). The average genetic distance to the closest relative shows a decrease with increase in sampling (Bergsten et al., 2012). Thus, our study which analyzed 14 (4 haplotypes) and 18 (7 haplotypes) individuals for *P. iranicus* and *P. patimari* Hg1, respectively, dropped the earlier estimate to 2.8%.

On page 29, Eagderi et al. (2020) state that “*P. patimari* is distinguished from *P. iranicus* by having six suborbital transverse rows (vs. seven rows)”, however, in the extended description of *P. patimari* on page 27, they confirm the presence of row *7*, incorrectly labeled as row *6*. In addition, based on the original description, one may infer that *P. patimari* is also distinguished from *P. iranicus* by absence of anterior oculoscapular pores ω and preopercular pores γ and ε (vs. present). Based on the examination of types and newly collected materials in this study, it became clear now the cephalic lateral line system of *P. patimari* was incorrectly described; we redescribed it in detail based on corrected morphological character states: infraorbital neuromast organs in seven transverse rows, four (*1*–*4*) before and three (*5s*, *6s* and *7*) above and two (*5i*, *6i*) below row *b*; AOC, POC and PC canals present, with pores *σ*, *λ*, *κ*, *ω*, *α*, *β*, *ρ* and *θ*, *τ* and *γ*, *δ* and *ε*, respectively. This is also the typical pattern of head lateral line system of *Ponticola* species, although many of these species still miss head lateral‐line system description and *P. syrman* has three transverse rows below hyomandibular row *b*, third one marked *5i*' as duplication of row *5i* in Miller (2003). This head lateral line system pattern is also common among all Neogobiini + Ponticolini clade (Zarei, Esmaeili, Schliewen, et al., 2021b) with some exceptions: *Mesogobius* spp. and *Neogobius bathybius* have infraorbital neuromast organs in 8–10 and 8 transverse rows, respectively. Intraspecific deviations in head lateral line pattern occur and are common among the Ponto‐Caspian gobiids, which therefore should not be taken as diagnostic features to describe new species. These deviations concern the following character states: (i) addition or deletion of pores in the course of canals [e.g., additional pores in the course of AOC or loss of pore *δ* in some specimens of *P. kessleri* (Ahnelt & Duchkowitsch, 2001), or presence of an additional pore between pores *κ* and *ω* in some specimens of *P. gorlap* from Sefidroud (Zarei, 2022)]; (ii) paired interorbital pores [e.g., paired pores *λ* in some specimens of *P. iranicus* from the upper Sefidroud sub‐basin (this study) or in some specimens of *P. kessleri* and *Proterorhinus* sp. (Ahnelt & Duchkowitsch, 2001)]; and (iii) presence of additional row before or below row *b* on one side of the head [e.g., in some specimens of *P. ratan* from Odessa (Pinchuk et al., 2003a), *P. hircaniaensis* (Zarei, Esmaeili, Kovačić, et al., 2022a), and *P. patimari* Hg3 from Shafaroud (this study)]. It was already found that between sister species of *Ponticola* genus there is no recognizable differences in the head lateral‐line system (Kovačić & Engin, 2008; Zarei, Esmaeili, Schliewen, et al., 2021b), and even the recent descriptions of new cryptic species among the entire *Gobius*‐lineage struggled to find differences to sister species or to similar congeneric species in the head lateral‐line system (e.g., Kovačić & Šanda, 2016).

Furthermore, based on our data, ranges for D2 branched rays (15–16, usually 16 vs. 15–17, usually 16), CPd (10.5–12.4 vs. 10.4–12.3% of SL), and Pl (22.6–27.6 vs. 25.8–30.0% of SL; in all cases, P reaches or passes vertical of D2 origin) are broadly overlapping for *P. iranicus* and *P. patimari* Hg1. Although present results have excluded morphological characters suggested by Eagderi et al. (2020) as diagnostic characters to distinguish between *P. patimari* and *P. iranicus*, our molecular and morphometric results (see below) do not dispute the validity of the former species. However, it has been pointed out that for the sister species or similar congeneric species supported by molecular data, additional effort should be invested in a search for useful diagnostic characters that can distinguish the species (Kovačić et al., 2021; Kovačić & Šanda, 2016).

**FIGURE 12:**
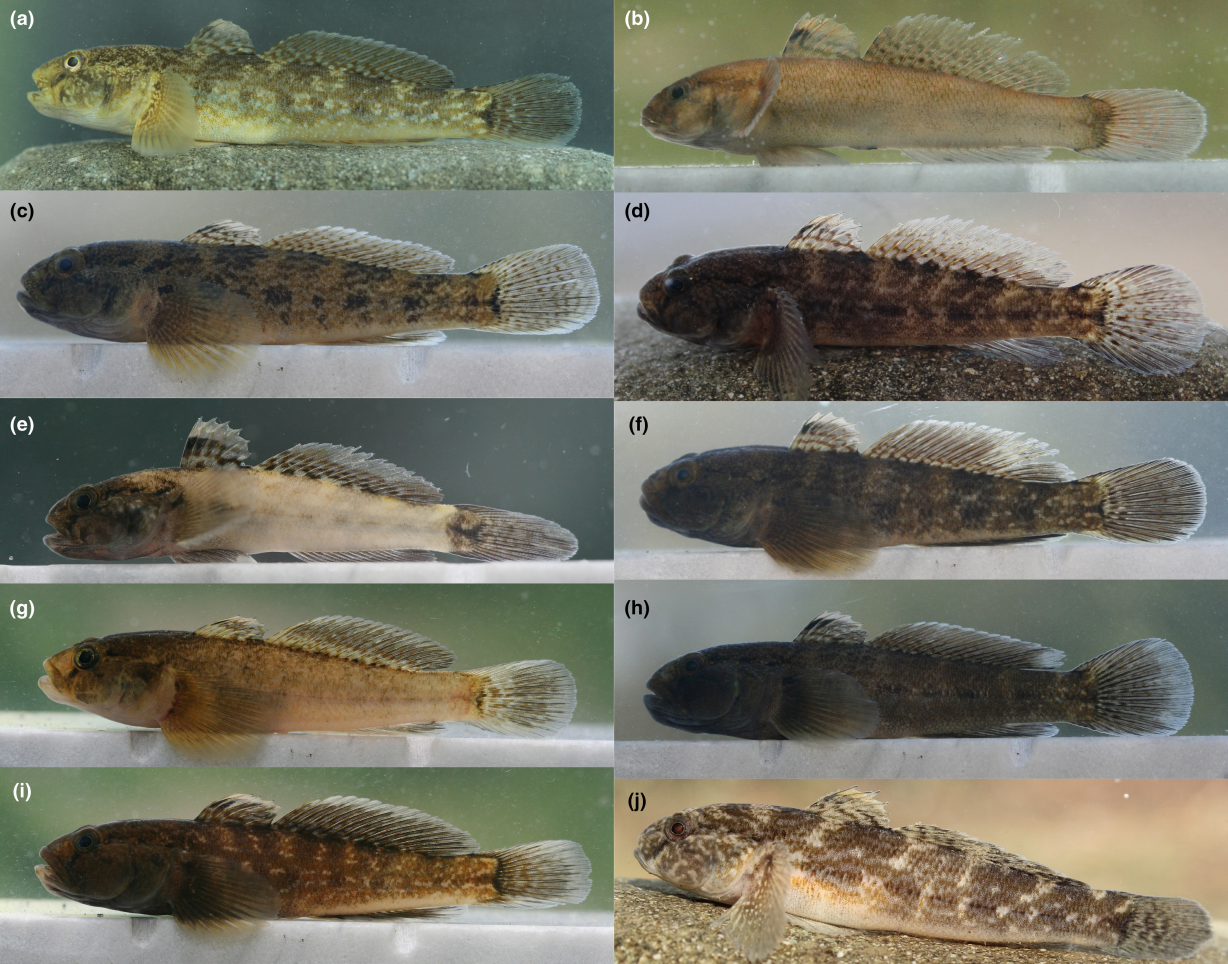
Intrapopulation and intraspecific coloration variability in *P. iranicus* collected from IM (a, b), and *P. patimari* collected from SH (c, d), SI (e, f), PO (g, h), and CH (i, j)

The morphological results only partly reinforced the indicated molecular phylogenetic subdivisions:

- The cephalic lateral line system pattern did not show interspecies differences supporting the results of mtDNA‐based phylogenetic work. As discussed above, it is already known that among gobiid species from large genera of *Gobius‐*lineage, like *Gobius* or *Ponticola*, some species share cephalic lateral line system so similar that it is hard or it is not possible to find the differences between species and express them as diagnostic characters (see species short descriptions and cephalic lateral line system pattern illustrations in Miller, 1986).
- The otolith shape analysis did not show congruence with the results of phylogenetic pattern. Otolith analysis has made significant contributions to the understanding of systematics and evolution of teleosts (e.g., Lombarte et al., 2018; Reichenbacher et al., 2007; Teimori et al., 2019). The otolith morphometric variables, inclination angles and classical shape descriptors showed that the otoliths of *P. iranicus* and *P. patimari* Hg1 are only slightly different in their rectangularity index. A comparative analysis of the extant Ponto‐Caspian gobiids also suggests only limited validation of this method for the assignment of otoliths to individual species within *Ponticola*; it can, however, sometimes support phylogenetic relationships and differentiation within several other genera (e.g., *Benthophilus* and *Neogobius*) and at higher taxonomic levels (Zarei, 2022). On the other hand, otolith outline analysis using wavelet transform showed notable phenotypic variation among samples not reflecting major phylogenetic subdivisions and spatial distributions of the main lineages. This results can be explained by the fact that the morphological attributes apart from certain intrinsic (phylo‐ and population genetic) factors may be influenced by additional extrinsic, e.g., environmental, effects (Charmantier & Garant, 2005; Klingenberg, 2010; Landaeta et al., 2019). A group of studies have concluded that otolith shape is genetically determined, often species specific and may mirror phylogenetic relationships (e.g., Lombarte & Castellón, 1991; Reichenbacher et al., 2009; Zhuang et al., 2015). However, it is now realized that it does not necessarily reflect genetic differences (e.g., Stransky, 2005; Vignon & Morat, 2010). Several studies suggest that while genetics constrain the overall shape of the otolith itself, environmental conditions such as food availability, depth, water temperature, salinity, substrate type, and exposure to environmental contaminants alter the rates of somatic and otolith growth, which in turn may affect otolith outline (e.g., Cardinale et al., 2004; Clark et al., 2021; Di Franco et al., 2019; Gagliano & McCormick, 2004; Hong et al., 2021; Lombarte & Lleonart, 1993; Oozeki & Watanabe, 2000; Schulz‐Mirbach et al., 2008; Vignon, 2018).
- The other qualitative characters, including coloration that has high intraspecific variability in these species (Figure 12), cannot distinguish between *P. iranicus* and *P. patimari*.
- PCA and DFA plots for the meristic and morphometric data, however, showed a clear separation of the two main subclades corresponding to *P. iranicus* and *P. patimari* (Hg1 + Hg2 + Hg3), suggesting the presence of significant morphological variation meriting formal taxonomic recognition. On the other hand, *P. patimari* Hg1 and *P. patimari* Hg2 + Hg3 presented a clear morphological continuum, which does not allow for practical taxonomic diagnosis. Thus, pending further investigation, we here adopt a conservative approach by considering the latter two groups to represent allopatric populations of *P. patimari*. Considering that other morphological and coloration characters cannot at the moment discriminate *P. iranicus* and *P. patimari* (present research; Eagderi et al., 2020), morphometric analysis of quantitative traits provides a proxy for morphological species recognition. This approach has been successfully applied in previous gobiid studies, allowing the delineation of morphologically similar species (Lima‐Filho et al., 2016; Neilson & Stepien, 2009b).

Accordingly, the present results revised distributional ranges of *P. iranicus* and *P. patimari*, narrowing known distributional range of *P. iranicus*, and expanding distributional range of *P. patimari* (Eagderi et al., 2020; Vasil'eva et al., 2015; Figures 1 and 4b).

According to Zarei, Esmaeili, Schliewen, et al. (2021b) and our molecular clock based estimates, the *P. iranicus* + *P. patimari* clade diverged from *P. syrman*, the only other species of the group distributed in inshore brackish water habitats of the Ponto‐Caspian seas, at ~0.9 Mya (95% HPD: 0.71–1.21 Mya; this study; Zarei, Esmaeili, Schliewen, et al., 2021b). Despite this recent divergence, this shift to a freshwater habitat may have led to body size evolution and noticeable morphological differences (see Santini et al., 2013; Seehausen & Wagner, 2014). *Ponticola iranicus* and *P. patimari* both differ from *P. syrman* in: (i) lower jaw slightly prognathous (vs. markedly prognathous); (ii) anterior membrane of pelvic disc with well‐developed and pointed lateral lobes (vs. with very shallow, rounded lateral lobes); (iii) having two infraorbital transverse rows below row *b* (vs. three rows below row *b*); (iv) coloration: highly variable (Figure 12), from yellowish grey to dark brown, dark/brown saddles on back, flanks with row of elongated dark/brown spots/bands along the midline, pectoral base with upper dark spot, D1 with light marginal band and dark anterior stripe (vs. pale grey, pectoral base lacking upper dark spot, D1 lacking marginal band and dark anterior stripe); (v) meristics: having less branched anal‐fin rays (10–13 vs. 13–15), and less scales in longitudinal scale rows (50–65 vs. 65–69); (vi) body size decrease with maximum total length 123.6 mm (vs. 280.0 mm), and (vii) body proportion changes: having deeper body depth at anal‐fin origin (17.5%–21.2% vs. 15.3%–17.7% SL), longer caudal peduncle (10.2%–12.4% vs. 7.7%–9.8% SL), longer anal‐fin spine (6.6%–8.9% vs. 5.5%–6.1% SL), longer eye diameter (17.8%–25.7% vs. 13.1%–14.7% HL), shorter cheek depth (15.2%–22.6% vs. 24.8%–29.3% HL), shorter lateral preorbital depth (10.4%–14.6% vs. 15.5%–19.5% HL), and shorter upper lip length (27.1%–39.7% vs. 40.2%–42.6% HL; Zarei, 2022).

### 4.2 Evolutionary history and genetic structure

Multiple glacial–interglacial cycles affected the Caspian Sea during the Pleistocene, causing periods of transgressions and regressions and extreme salinity oscillations (Chepalyga, 1984; Dumont, 1998; Zenkevich, 1963). Present results suggest that the climatic oscillations of the late Early, Middle and Late Pleistocene were associated with the cladogenesis within the *P. syrman group*. Names for the historic stages of the Caspian Sea basin are used *sensu* Krijgsman et al. (2019). The strong Caspian Tyurkyanian (= Turkanian) regression (1.05–0.95 Mya, ~150 m b.s.l. for ~0.1 Myr) at the boundary of the Apsheronian and Bakunian ages was caused by the abrupt decrease in atmospheric precipitation and river flow due to global climatic cooling and formation of continental glaciation in the Volga River basin, Russian plain, Urals and mountain valley glaciers in the Caucasus (Klenova et al., 1962; Krijgsman et al., 2019; Svitoch, 2012). During this phase of high salinity and reduced habitat availability, an arid climate prevailed in the south Caspian region (Abdullayev, 2000; Tagieva et al., 2013; Zastrozhnov et al., 2020), and rivers draining into the Caspian Sea may have become seasonal or dry for short periods (Sands et al., 2019). According to our molecular clock based estimates, brackish water ancestors of the contemporary *P. iranicus* + *P. patimari* diverged from *P. syrman* ca. 0.9 Mya: (i) trapped in freshwaters of the upper Sefidroud sub‐basin when sea‐level dropped from ~+70 to ~−150 m, or (ii) invaded headwaters of the Sefidroud River (possibly, as a result of the high salinity of the Caspian Sea) and subsequently adapted to freshwater conditions. The ancestor of *P. iranicus* + *P. patimari* adapted to freshwater, while “marine” *P. syrman* adapted to increased salinity of the Caspian Sea in the Tyurkyanian period, so the ecological gap and isolation between them also increased. A similar effect has been outlined for evolution of several other aquatic species in the south Caspian basin (e.g., Sands et al., 2019), including the radiation of *Ponticola hircaniaensis* (Zarei, Esmaeili, Kovačić, et al., 2022a) and a possible bottleneck in *Proterorhinus nasalis* (Zarei, Esmaeili, Schliewen, & Abbasi, 2022b). Evolution in south Caspian refugia have been also reported for several species in other groups, e.g., rock lizards of the genus *Darevskia*, Arribas, 1999 (Ahmadzadeh et al., 2013; Saberi‐Pirooz et al., 2018), the Caspian green lizard *Lacerta strigata* Eichwald, 1831 (Saberi‐Pirooz et al., 2021), the Caucasian pit viper *Gloydius halys caucasicus* (Nikolsky, 1916) (Asadi et al., 2019), the Anatolian mountain frogs of the genus *Rana* Linnaeus, 1758 (Veith et al., 2003), and the freshwater crab *Potamon ibericum* (de Bieberstein, 1808) (Parvizi et al., 2018).

The Tyurkyanian regression was followed by the Bakunian sea‐level high stand up to about 50 m a.s.l. and a temporal extent of ca. 378–480 kya (Krijgsman et al., 2019), which was the largest transgression in the Pleistocene history of the Caspian Sea. The Bakunian Sea became considerably desalinated, and a warmer and humid climate persisted during this period (Abramova, 1974; Filippova, 1997; Svitoch et al., 1998; Yakhemovich et al., 1986). It is likely that the Bakunian increase in climatically suitable habitats as well as in precipitation and freshwater flow had caused habitat connectivity during this period, leading to downstream expansion into the eastern and western sub‐basins and to a further radiation within the freshwater clade; according to this scenario, (i) first, separation of the pre‐*iranicus* and pre‐*patimari* clades in the early Bakunian; the population that remained the headwaters of the Sefidroud sub‐basin eventually evolved into *P. iranicus*, (ii) divergence of the pre‐*patimari* Hg1 and pre‐*patimari* Hg2 + Hg3 took place in the middle Bakunian, and (iii) separation of the pre‐*patimari* Hg2 and pre‐*patimari* Hg3 developed in the late Bakunian. Subsequently, the Hg2 and Hg3 groups underwent further expansion and radiation within the central and western sub‐basins during the early Khazarian stage. Our phylogeographic assessments suggest a transitional zone between these two groups occurring in the region of the lower Sefidroud River (i.e., KY). Therefore, we propose that Hg2 and Hg3 mainly occur east and west of the Sefidroud River, respectively. Finally, during the early Khazarian phase, subsequent diversifications of *P. iranicus* and *P. patimari* in the colonized rivers took place, which produced a bush‐like genealogy characterised by large numbers of private haplotypes and rare cases of shared haplotypes (e.g., H16 in *P. patimari*). This pattern suggests that contemporary connectivity and gene flow between catchments (compared with the Bakunian stage) is limited.

### 4.3 Conservation units and management propositions

The south Caspian freshwater habitats have been and continue to be threatened by the main causes of biodiversity loss (Esmaeili et al., 2015; Mousavi‐Sabet, 2021). In this context, a careful and accurate taxonomic assessment of their ichthyofauna is a prerequisite for sketching effective conservation measures. The taxonomic revision of the *P. syrman group* has several implications for conservation: (i) it confirms the presence of two valid freshwater species as the main units of conservation, (ii) it revises and documents a narrow distributional range and diversity for *P. iranicus*, but a wider distributional range and diversity for *P. patimari*, and (iii) it allows for delimiting conservation units below species level, i.e., evolutionary significant units (ESUs) and management units (MUs) [see Fraser & Bernatchez, 2001, and Allendorf et al., 2013 for complete definitions], so that limited conservation resources can be utilized optimally (Ryder, 1986).

Reciprocal monophyly in mtDNA, high estimates of genetic divergence, lack of gene flow, distinct spatial distributions, and morphometric differences suggest long‐term isolation between the three genetic clusters, *P. iranicus*, *P. patimari* Hg1, and *P. patimari* Hg2 + Hg3, which are sufficiently differentiated to be considered ESUs.

ESUs may themselves be sub‐structured at hierarchical levels generally recognized as MUs (sensu Moritz, 1994), another logical units for conservation efforts (Moritz, 1994). The *P. patimari* Hg2 + Hg3 ESU is comprised of two geographically structured subgroups, Hg2 and Hg3, with a contact zone in the lower Sefidroud sub‐basin. The molecular and morphometric data indicate that individuals from the Hg2 and Hg3 subgroups comprise just one biological group; however, this *P. patimari* Hg2 + Hg3 ESU is best viewed as comprising two MUs, Hg2 and Hg3, according to the original definition of Moritz (1994). *Ponticola patimari* has a high level of genetic diversity if treated as only one panmictic group, however, species structured into ESUs and MUs, necessitate separate monitoring and management of each unit (Frankham et al., 2002).

The three ESUs are each subject to different and distinct threats stemming from differences between the area occupied by each lineage and levels of habitat degradation within these areas; thus, they are natural candidates for differentially optimized management strategies. *Ponticola iranicus* is particularly vulnerable as it is composed of a single ESU with a narrow distributional range associated with low mtDNA variability which makes it more sensitive to extrinsic changes and therefore a greater risk of extinction (Frankham et al., 2002). The Sefidroud Dam (= Manjil Dam), constructed upstream of the Sefidroud River to store water for irrigation and produce hydroelectric power, is the main threat to *P. iranicus*. The *Ponticola patimari* Hg2 + Hg3 ESU features a broad geographical distribution from PO to KA. From a conservation perspective, this ESU possesses relatively high levels of genetic diversity indicating that the population does not require immediate conservation attention. In addition, the distribution range of this ESU harbours many available habitats for the species to occupy. The *P. patimari* Hg1 ESU, despite having wider distributional range, shows levels of mtDNA variability similar to the *P. iranicus*, most likely biased by poor sampling at TO, NO, and KH.

In Iran, temperature will rise and precipitation will decrease under the climate change (Dastorani & Poormohammadi, 2016; Rahimi et al., 2018). These changes will affect freshwater habitats and will make them less habitable for freshwater species. Modeling the impacts of climate change on distribution of *P. iranicus sensu* Vasil'eva et al. (2015) predicts that the species will significantly lose its suitable habitats in the upper Sefidroud sub‐basin over the next few decades which may lead to the range reduction or range shift in *P. iranicus sensu stricto*, while suitable habitats will increase in the eastern regions which may lead to the range expansion of the *P. patimari* Hg1 ESU (Yousefi et al., 2020).

## 5 COMPARATIVE MATERIAL

*Ponticola cyrius*: PMR VP1691, 3 specimens, 86.5–88.7 mm SL, Turkey, Hanak, Kura River, S. Engin & D. Turan, 19 June 2003.

*Ponticola goebelii*: ZM‐CBSU S014‐1, 1 specimen, 55.4 mm SL, Iran, Gilan prov., Anzali beach, southern Caspian Sea, 37°28'21.0"N 49°31'05.5"E, K. Abbasi, 18 November 2002.—Data (Caspian Sea: Azerbaijan, Iran, Dagestan, and Turkmenistan: 7 specimens, 73.0–165.0 mm SL) from Pinchuk et al. (2003a)—Data from Bogutskaya et al. (2013).

*Ponticola gorlap*: ZM‐CBSU S093‐1–2, 2 specimens, 87.9–118.2 mm SL, Iran, Gilan prov., Sefidroud at Imamzadehashem, southern Caspian Sea basin, 37°01'23.9"N 49°37'56.7"E, M. Masoudi, M. Mehraban & R. Khaefi, 17 July 2014.—ZM‐CBSU S094‐1–2, 2 specimens, 83.6–103.5 mm SL, Iran, Gilan prov., Chalvan River, southern Caspian Sea basin, 38°17'34.5"N 48°52'33.4"E, H.R. Esmaeili, S. Vatandoust & R. Khaefi, 19 June 2012.—ZM‐CBSU S094‐3–5, 3 specimens, 79.8–97.2 mm SL, Iran, Gilan prov., Chalvan River, southern Caspian Sea basin, 38°17'34.5"N 48°52'33.4"E, Masoudi, M. Mehraban & R. Khaefi, 30 June 2014.—ZM‐CBSU S095‐1, 1 specimen, 107.2 mm SL, Iran, Gilan prov., Chalvan River, southern Caspian Sea basin, 38°17'34.5"N 48°52'33.4"E, F. Zarei & Y. Bakhshi, 26 August 2019.—ZM‐CBSU S078‐1–3, 3 specimens, 83.9–101.4 mm SL, Iran, Gilan prov., Anzali Wetland, southern Caspian Sea basin, 37°28'04.8"N 49°21'14.6" E, K. Abbasi, 06 July 2017.—ZM‐CBSU S067‐1–2, 2 specimens, 162–187 mm SL, Iran, Mazandaran prov., Neka Beach, north of Tejen Lateh, southern Caspian Sea, 36°49'37.3"N 53°10'26.7"E, F. Zarei & R. Sadeghi, 15 November 2018.—ZM‐CBSU S096‐1–2, 2 specimens, 80.6–102.2 mm SL, Iran, Gilan prov., Siahdarvishan River, southern Caspian Sea basin, 37°21'09.6"N 49°25'17.0"E, F. Zarei & Y. Bakhshi, 27 August 2019.—ZM‐CBSU S097‐1–3, 3 specimens, 90.7–96.3 mm SL, Iran, Gilan prov., Sefidroud at Kuchesfahan, southern Caspian Sea basin, 37°14'46.3"N 49°49'03.5"E, F. Zarei & Y. Bakhshi, 28 August 2019.—ZM‐CBSU S098‐1–2, 2 specimens, 85.4–89.2 mm SL, Iran, Mazandaran prov., Neka River, southern Caspian Sea basin, 36°37'52.0"N 53°20'29.0"E, F. Zarei, 1 October 2019.

*Ponticola hircaniaensis* (all from Kaboudval Stream, Golestan prov., Iran): ZM‐CBSU S099‐1 to S099‐12, 12 specimens, 52.8–80.8 mm; Y. Bakhshi, Z. Ganjali & A. Jouladeh‐Roudbar, 28 August 2017.—ZM‐CBSU S100‐1 to S100‐4, 4 specimens, 49.8–95.9 mm; F. Zarei & Y. Bakhshi, 31 August 2019.—ZM‐CBSU S101‐1 to S101‐15, 15 specimens, 54.9–91.3 mm; F. Zarei, 27 August 2021.

*Ponticola syrman*: ZM‐CBSU S065.2‐1, 1 specimen, 183.2 mm SL, Iran, Mazandaran prov., Neka beach, southern Caspian Sea, 36°49'43.8"N 53°10'24.0"E, F. Zarei & R. Sadeghi, 15 November 2018.—ZM‐CBSU S092‐1–6, 6 specimens, 171.2–230.0 mm SL, Iran, Gilan prov., Anzali beach, southern Caspian Sea, 37°28'21.0"N 49°31'05.5"E, K. Abbasi.

## AUTHOR CONTRIBUTIONS

**Fatah Zarei:** Conceptualization (lead); Data curation (lead); Methodology (lead); Investigation (lead); Formal Analysis (lead); Software (lead); Visualization (lead); Writing – original draft (lead); Writing – review & editing (lead). **Hamid Reza Esmaeili**: Conceptualization (supporting); Data curation (supporting); Resources (lead); Supervision (lead); Writing – review & editing (supporting). **Reza Sadeghi:** Data curation (supporting); Formal Analysis (supporting); Writing – review & editing (supporting). **Ulrich K. Schliewen:** Data curation (supporting); Supervision (supporting); Writing – review & editing (supporting). **Marcelo Kovačić:** Data curation (supporting); Writing – review & editing (supporting). **Keyvan Abbasi:** Data curation (supporting); Writing – review & editing (supporting). **Ali Gholamhosseini:** Software (supporting); Supervision (supporting); Writing – review & editing (supporting).

## CONFLICT OF INTEREST

We declare no conflict of interest.

## Supporting information


Tables S1–S7

## Data Availability

The COI sequences, otoliths, and specimens are deposited in GenBank and ZM‐CBSU, respectively.
